# The Application of Technological Intervention for Stroke Rehabilitation in Southeast Asia: A Scoping Review With Stakeholders' Consultation

**DOI:** 10.3389/fpubh.2021.783565

**Published:** 2022-02-07

**Authors:** Siti Nur Suhaidah Selamat, Rosalam Che Me, Husna Ahmad Ainuddin, Mazatulfazura S. F. Salim, Hafiz Rashidi Ramli, Muhammad Hibatullah Romli

**Affiliations:** ^1^Department of Industrial Design, Faculty of Design and Architecture, Universiti Putra Malaysia, Seri Kembangan, Malaysia; ^2^Malaysian Research Institute on Ageing, Universiti Putra Malaysia, Seri Kembangan, Malaysia; ^3^Department of Rehabilitation Medicine, Faculty of Medicine and Health Sciences, Universiti Putra Malaysia, Seri Kembangan, Malaysia; ^4^Centre of Occupational Therapy Studies, Faculty of Health Sciences, Universiti Teknologi MARA Selangor, Shah Alam, Malaysia; ^5^Department of Rehabilitation Medicine, Hospital Pengajar, Universiti Putra Malaysia, Seri Kembangan, Malaysia; ^6^Department of Electrical and Electronic Engineering, Faculty of Engineering, Universiti Putra Malaysia, Seri Kembangan, Malaysia

**Keywords:** technological intervention, rehabilitation, developing countries, low-middle income countries, developed countries, Southeast Asia, cerebrovascular accident

## Abstract

**Background:**

The technological intervention is considered as an adjunct to the conventional therapies applied in the rehabilitation session. In most high-income countries, technology has been widely used in assisting stroke survivors to undergo their treatments. However, technology use is still lacking in Southeast Asia, especially in middle- and low-income countries. This scoping review identifies and summarizes the technologies and related gaps available in Southeast Asia pertaining to stroke rehabilitation.

**Methods:**

The JBI manual for evidence synthesis was used to conduct a scoping study. Until September 2021, an electronic search was performed using four databases (Medline, CINAHL, Scopus, ASEAN Citation Index). Only the studies that were carried out in Southeast Asia were chosen.

**Results:**

Forty-one articles were chosen in the final review from 6,873 articles found during the initial search. Most of the studies reported the implementation of technological intervention combined with conventional therapies in stroke rehabilitation. Advanced and simple technologies were found such as robotics, virtual reality, telerehabilitation, motion capture, assistive devices, and mobility training from Singapore, Thailand, Malaysia, and Indonesia. The majority of the studies show that technological interventions can enhance the recovery period of stroke survivors. The consultation session suggested that the technological interventions should facilitate the needs of the survivors, caregivers, and practitioners during the rehabilitation.

**Conclusions:**

The integration of technology into conventional therapies has shown a positive outcome and show significant improvement during stroke recovery. Future studies are recommended to investigate the potential of home-based technological intervention and lower extremities.

## Introduction

Strokes or a cerebrovascular accident (CVA) is caused by the blockage or bursting of the cerebral blood vessels—the leading cause of neurological disorder globally (1). Stroke is one of the most common non-communicable diseases worldwide, especially in Asia (2, 3). In high-income regions of Asia Pacific, North America, East, and Southeast Asia, those aged 50–64 years have the highest prevalence rates of both ischemic and hemorrhagic stroke (2). According to the World Health Organization (WHO), stroke or cerebrovascular accidents are the second leading cause of death and the third leading cause of the disabilities (3–5). Aldehaim et al. (6) also stated that 40% of those who survived a stroke experience a physical disability that needs special treatment, and another 10% of them end up in a nursing home or other long-term rehab centres. Caring for stroke survivors can be depressing and burdensome, and it may affect the well-being of both survivors and caregivers.

The Southeast Asia region with the size of over 4.5 million km^2^ constitute of low-income (Myanmar, East Timor, Cambodia, Laos), middle-income (Vietnam, Philippines, Indonesia, Thailand, Malaysia), and high-income (Brunei, Singapore) countries (2–7). The region is heavily populated with a population of nearly 700 million people and the area is diverse in terms of ethnicity and culture, but it also shares similarities in dietary, climate, and lifestyle (2, 3, 8). In terms of stroke prevalence, Indonesia is at 8.0, the Philippines is at 9.0, Singapore is 36.5 (>45 years old), Thailand (18.8 for >45 years old), Vietnam (6.1), and Malaysia (7.0) for every 1,000 population (2). Stroke is a major public health problem because it is the predominant cause of physical impairment and disability among adults (9).

Stroke recovery treatment or rehabilitation typically requires conventional therapy, where it is labor intensive involving therapist-clients education and training (10). Rehabilitation is a goal-oriented process that helps people with disabilities reach their full potential in emotional, physical, cognitive, social, and functional skills (11, 12). Rehabilitation physicians, occupational therapists, physiotherapists, speech therapists, rehabilitation nurses, and medical social workers are among the healthcare professionals involved (13, 14). Generally, the rehabilitation process may take place in various settings, including inpatient, outpatient, group, and home-based (15–18). The survivor's period of recovery from stroke depends on the stroke's severity stage, and usually, the treatment starts once the main cause of stroke has been diagnosed (13).

The rehabilitation treatment is essential for stroke survivors to achieve the highest level of functional independence, reducing or preventing the impairments (11). However, this process could be time-consuming and will lead to extra costs. The cost is described as medical procedure on ancillary or referral and an idea of loss of productivity and the costs generated from the recovery processes (8). This fact has become a critical issue to both health practitioners and clients. Thus, due to these scenarios, technological solutions could be the beneficial adjunct and alternative toward the existing conventional method or therapy, making it more accessible to everyone. In addition, the technological intervention provides access to rehabilitation services for those facing physical, financial, and attitudinal barriers.

Most developed countries implement the technology in rehabilitation therapy as an initiative to help stroke survivors during their recovery period other than focusing only on conventional therapy (19, 20). It is known that the recovery process can be varied depending on several factors such as the client's impairment level, the therapy intensity and access, or the individual activity and participation. Technological interventions such as robotics, video-based therapy, teleconferencing, virtual reality, and assistive devices are more commonly explored and utilized in developed countries, mainly due to their readiness and availability (21, 22).

However, the extent of technology use in low-and middle-income countries is uncertain as it is not explored comprehensively. Therefore, this scoping review aims to systematically identify and review the evidence of using technological intervention combined with conventional therapy or traditional methods in helping the recovery period of stroke survivors. The efficacy of rehabilitative interventions is investigated and discussed according to technological clusters or categorizations.

## Methodology

The framework consists of seven consecutive stages following the Joanna Briggs Institute (JBI) framework (23): (i) developing the review question, (ii) defining inclusion and exclusion criteria, (iii) conducting a search strategy, (iv) evidence screening and study selection, (v) data extraction, (vi) data analysis, and (vii) presentation of results. Each stage is discussed further in the following subtopics, and the Preferred Reporting Items for Systematic Reviews and Meta-analysis extension for scoping reviews (PRISMA-ScR) (24) (Supplementary Table 1) was adopted as a guideline for the report of the scoping review.

### Developing a Review Question

The study adopted the Population-Concept-Context (PCC) framework (25) to determine the research question's extent. The population is stroke survivors, and the concepts are the implementations of the technological intervention in stroke rehabilitation. The context of this study is focused on the Southeast Asia region. This scoping review was developed based on the question, “What are the technologies and gaps available in Southeast Asia pertaining to stroke rehabilitation?”

#### Defining Inclusion and Exclusion Criteria

Studies considered to be included in this scoping review when they fulfill the following criteria:

(i) Stroke rehabilitation as defined by Young and Forster (26) as using a mixture of a therapeutic and problem-solving approach to limit the impact of stroke-related brain damage on daily life,
(ii) Utilizing technology as a medium of therapy or rehabilitation delivery. Technology is a broad term that refers to how one uses and understands the instruments and craft and how it affects the ability to control and adapt to the social and physical environment. It can also refer to physical objects that people use, such as computers, hardware, or utensils, but may also lead to broader themes such as structure, organizational methods, and techniques. Meanwhile, rehabilitation technology uses technology to meet the needs of people with disabilities. It helps cut the barriers and gives more opportunity to people with disabilities in education, rehabilitation, employment, living at home, and recreation.
(iii) The technology investigated is either for the stroke survivors or people surrounding them (i.e., family members, caregivers) or anybody that deals with the stroke cases,
(iv) The study is conducted in Southeast Asia, and
(v) Any study design (i.e., case study, qualitative study, quantitative survey, experiment) and setting (i.e., clinical, community, institution) are eligible to be included.

Meanwhile, exclusion criteria are as follows: (i) Non-English, (ii) gray literature (i.e., thesis, dissertation, book), (iii) non-original or review study (i.e., letter to editor, literature review, protocol), and (iv) no full text available (i.e., conference abstracts). The exclusion of non-English and gray literature is expected to have minimal impact on the findings (27, 28).

#### Conducting a Search Strategy

The electronics search was conducted using the following databases: MEDLINE, CINAHL, Scopus, MyCite, and ASEAN Citation Index on 30th November 2019 and last updated on 22nd September 2021. The keywords used are related to strokes and its associated terminologies (e.g., cerebrovascular accidents, CVA), rehabilitation (including physiotherapy, occupational therapy, speech therapy, etc.), and Southeast Asia (including each name of the countries members). Boolean operators, parenthesis, exact, and wildcards were used when necessary. Search string used was (“cerebrovascular accident” OR “CVA” OR “stroke”) AND (“rehabilitation” OR “therapy” OR “therap^*^” OR “occupational therapy” OR “physiotherapy” OR “physical therap^*^” OR “speech therap^*^”) AND (“Southeast Asia” OR “Malaysia” OR “Singapore” OR “Thailand” OR “Indonesia” OR “Brunei” OR “East Timor” OR “Cambodia” OR “Myanmar” OR “Vietnam” OR “Laos” OR “Philippines”) on those four databases.

#### Evidence Screening and Study Selection

The titles and abstracts were independently reviewed by two authors (SNSS and HAA), and the eligibility of the studies for inclusion was based on the previously mentioned criteria. Any conflicts between the two authors were resolved through discussions for each article. Studies were included in the first screening stage (titles plus abstract) if any of the two authors agreed that they were eligible for inclusion or if there was a dispute about whether to exclude them. Studies were included in the second screening stage (full text) when both authors agreed to match all the inclusion criteria. When contradictory studies were detected during the full-text screening, an independent arbitrator (MHR) an author with a healthcare background, was consulted. The two authors' pre-consensus agreement on the included full-text articles was calculated using percentages. Because a critical appraisal of each study is not required for scoping reviews, no quality evaluation was carried out.

#### Data Extraction

The summary of all the study details, including the citation, nation, study objective, design, setting, interventions, and findings, was presented in a matrix table. The technological intervention was then classified either as advanced or simple.

#### Data Analysis

The findings obtained from the review were summarized into observational studies, intervention studies, and qualitative studies. The review syntheses were integrated with the stakeholders' consultation session described extensively in the next section. All the data on the efficacious and validity of using the technological intervention in stroke rehabilitation in Southeast Asia was documented.

#### Consultation

As part of the data analysis, stakeholders were invited to engage in a roundtable discussion or known as consultation. The discussion-like exercise aims to identify stakeholders' priorities and questions to guide the literature review (2). The session with the stakeholders mimics a qualitative research design on FGD session (29) but could be not rigorous as it is a complementary step in a scoping review (30). Six stakeholders were recruited from various fields to provide richness and expertise contribution. Prior to the discussion, all the participants gave their informed consent. The six stakeholders chosen were an industrial designer (*n* = 1), an ergonomist (*n* = 1), an engineer (*n* = 1), a physiotherapist (*n* = 1), an occupational therapist (*n* = 1) and a physiatrist (*n* = 1). The consultation session was done virtually via the Zoom teleconference platform. Each participant was given a summary of the preliminary findings of this scoping review and a set of open-ended questions to guide the discussions; (i) What is the current practice of technology-use intervention compared to the gathered literature? (ii) What is the perception of the efficacy of technologies used in stroke rehabilitation? (iii) What is the perception of implementing technologies as a medium of intervention for stroke rehabilitation? and (iv) What improvements and suggestions can be provided for technology-based application for stroke rehabilitation intervention? The discussion was conducted in pidgin languages which were convenient to the participants. The whole session was recorded using a voice recorder and through note-taking. The qualitative data were analyzed by developing a coding excerpt from the session. The themes were generated and selected by comparing with review findings and discussions among the authors. One session was conducted and required two and a half hours to complete.

#### Presentation of the Results

The findings were reported narratively, and the literature and stakeholders' consultation information were synthesized. The narrative review is sequenced in themes generated among the authors. The result is combined with external literature—not only from the systematic searching inclusion, to enhance the understanding and synthesis.

## Results

**Figure 1:**
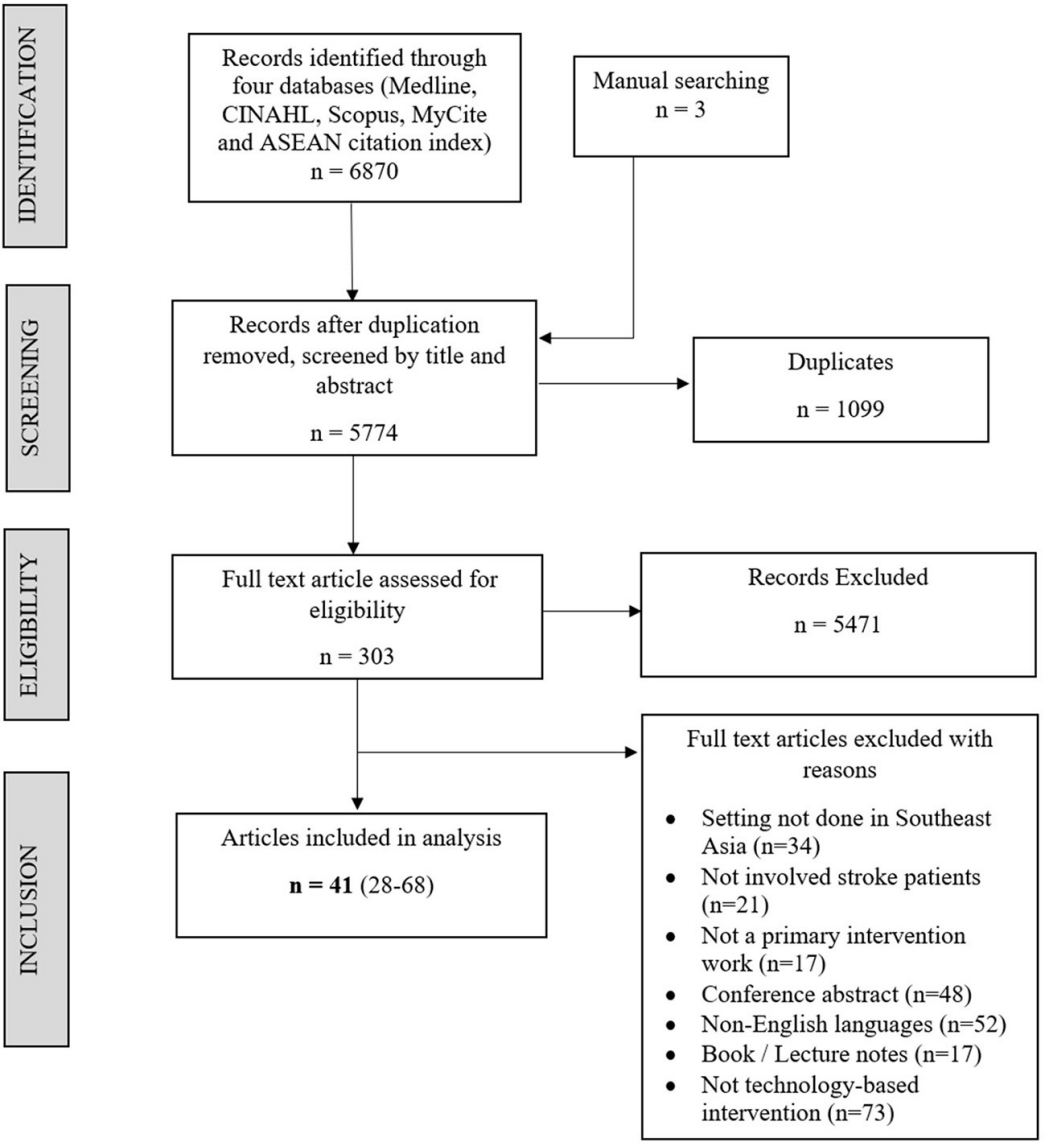
Screening process flowchart.

The initial search yielded a total of 6,873 citations from five electronic databases, and three additional manually founded citations based on Figure 1. The reasons for excluding the articles during the full-text screening are provided in Figure 1. From the screening process, a total of 41 studies (31–71) met the eligibility requirements and were included in this scoping analysis, as summarized in Supplementary Table 1.

A total of 1,470 participants participated from the selected 41 studies with different impairments, types, and stages of stroke severity. The data was narratively summarized according to a pre-defined theme on survivors' diagnosis, rehabilitation intervention available in recovery sessions, and stroke survivors' intervention. The theme was established through discussions among researchers, who compared the findings of various studies. The review's findings were combined with the stakeholders' consultation support.

### Characteristics of Included Studies

The studies found were in the following countries: Singapore (*n* = 25) (31, 34, 35, 38, 39, 42, 44–46, 48–54, 56–58, 64–69), Thailand (*n* = 10) (32, 33, 36, 41, 43, 47, 51, 59, 61, 63), Malaysia (*n* = 5) (37, 40, 55, 68, 71), Indonesia (*n* = 1) (62) and no study were found from Myanmar, Vietnam, Philippines, Laos, Brunei, East Timor and Cambodia. All the included studies were written in the English language. The studies were published between 2009 until 2021. From the 41 articles, there are randomized-controlled studies (*n* = 18), clinical studies (*n* = 7), case report (*n* = 2), an experimental study (*n* = 2), a crossover study (*n* = 1), a pilot study (*n* = 7), an open-labeled study (*n* = 2) and a qualitative study (*n* = 2). Thirty-two studies (*n* = 32) were conducted in the hospitals and the remaining nine (*n* = 9) were conducted in the community.

### Types of Technological Intervention in Southeast Asia

The most complex or newest technology accessible in post-stroke rehabilitation can also be referred to as advanced technological intervention (72, 73). Simple technology is referred to as traditional or non-mechanical, such as crafts and tools that pre-date the Industrial Revolution 4.0 concept (74–77). The simple technology can be practiced or fabricated with a minimal capital investment by an individual, and a single individual's knowledge of the practice can be comprehended (77).

### Advanced Technological Intervention

The study has classified six (*n* = 6) types of advanced technological intervention for post-stroke rehabilitation, which are robotics, transcranial magnetic stimulation (TMS), transcranial direct current stimulation (tDCS), motion analysis, motion capture, and virtual reality.

#### Robotics

Modern robotics have made tremendous progress and contribution to healthcare as they can help physicians perform various tasks (74–80). Robotic adoption is increasing tremendously in hospitals—robotics help regain and improves the function in both upper and lower extremities. In this scoping review, seven (*n* = 7) studies (31, 35, 42, 45, 47, 62, 67) were identified that implemented the robot-assisted therapy intervention in helping the survivors regain their abilities to the highest level of independence. Robotics technology found in the literature include: MIT-Manus robot coupled with EEG-based MI-BCI, a combination of EEG-based MI-BCI Haptic Knob robotic rehabilitation, a robotic exoskeleton with EMG signal, a Haptic Knob rehabilitation robot, and a soft robotic glove both focuses on grasping assistance. All the robotics mentioned before focus on upper extremities rehabilitation. From the studies mentioned, six (*n* = 6) studies (31, 35, 42, 45, 47, 67) showed that the use of robotics was effective in helping stroke survivors, while only Utomo et al. (62) reported that the use of the robotic is not effective for short-term rehabilitation. Medical robots are extremely useful in assisting physicians. However, being professionally trained to work with the medical robots and for the robot to fully respond to the clinician's instructions are somewhat time-consuming and require continuous training (81).

#### Transcranial Magnetic Stimulation

In the TMS method, magnetic impulses were sent through the skull to stimulate the brain. The treatments have shown promising results in improving the upper extremities in stroke survivors (82, 83). Three (*n* = 3) studies (41, 51, 60) included in this scoping review reported the use of TMS as a treatment to help stroke survivors. Most of the survivors who completed the entire course of this treatment experienced an improvement in their impairments after 6 months of treatment (51). The treatment used was able to help the stroke survivors to recover their movement and brain function. All the studies included here implemented this technology to treat upper-limb impairment (shoulder to hand). The results show that TMS may enhance the paretic arm reach-to-grasp performance on the non-lesioned hemisphere. From the studies mentioned, it is found that two (*n* = 2) studies (41, 51) had shown that using the TMS method is helpful for stroke survivors. Although most of the studies had shown the effectiveness of using the TMS method, a study from Tretriluxana et al. (60) shows that this intervention is only applicable toward the smaller objects when using the reach-to-grasp (RTG) action.

### Transcranial Direct Current Stimulation

From the past three decades, tDCS has become an increasingly popular technique in rehabilitation treatment (84). The use of tDCS in stroke research has gained particular interest, because both online and offline effects of tDCS can improve functional outcomes (85, 86). In the tDCS technique, a mild electrical current travel through the skulls and stimulates the brain. This treatment can help the survivors recover their movement from stroke and other conditions. Seven (*n* = 7) studies (38, 46, 56, 59, 63, 69, 71) in this scoping review found that this technique did improve the recovery process. Majority (38, 46, 56, 63, 71) showed positive results in both upper and lower limb functions, except two (*n* = 2) studies (59, 69). These two studies reported that tDCS does not increase the motor activity on lower limb muscles and gait performance and does not improve any motor function in stroke survivors.

#### Motion Analysis [Motor Imagery Brain-Computer Interface (MI-BCI), Electroencephalogram (EEG), and Electrocardiograms (ECG)]

Motion analysis captures the video of the human motion with specialized computer software that analyses the motion in detail. This technique provides the healthcare practitioner with a detailed picture of a person's specific movement challenges to guide proper therapy. In this scoping review, nine (*n* = 9) studies (38, 39, 45, 46, 48, 56, 62, 64, 69) implemented this treatment with stroke survivors. Most studies (39, 45, 46, 56, 64) have shown a tremendous enhancement on the upper limb rehabilitation treatment with this technique, except for two studies (62, 69). The intervention is less effective for short-term improvement on lower-limb function (62), similar to the single conventional intervention (69).

#### Motion Capture

Motion capture is a technique that involves using technology capable of recording and determining the location of points on an actor's body in a particular amount of time (87). A motion capture device is required to precisely monitor participants' movement, to allow physicians to deliver therapeutic advice based on observable data (88). It typically occurs by using a costume or other similar device, which the actor should dress him or herself (87). Only three (*n* = 3) studies (34, 50, 70) included in this scoping review used motion capture as a strategy for stroke recovery, such as Nintendo Wii. All the studies mentioned above show that motion capture in the rehabilitation setting could enhance the conventional therapy in upper limb subacute and chronic stroke survivors. The studies showed much potential as an adjunct therapy in upper limb stroke recovery and can be employed effectively in an acute inpatient environment.

#### Virtual Reality

Virtual reality has emerged as a therapeutic tool facilitating motor learning for balance and gait rehabilitation (89–91). The survivors can retrain themselves to perform the motions within a virtual environment (92). The use of VR-based therapy could provide a positive learning experience while at the same time could be engaging and motivating. Besides, VR applications have a huge potential for education and training purposes since the simulation can be conducted repeatedly which could not be replicated in a real-world application (93). This offers a significant advantage over conventional training. From the included studies in this scoping review, seven (*n* = 7) studies (39, 40, 44, 50, 53, 55, 57) implemented VR intervention combined with the conventional method for the rehabilitation treatment. Five (*n* = 5) studies demonstrate that the combined intervention has improved both upper limb function and lower limb postural standing balance, while only two (*n* = 2) studies (40, 53) show the opposite result. Both studies stated that this technology does not necessarily enhance physical function or improve the upper extremity recovery in stroke survivors.

### Simple Technological Intervention

Simple technological intervention can also refer to a system or equipment that is non-battery powered and relatively easier to fabricate (61). This technology is affordable, adaptable, easily managed, and only uses little energy and resources to stay entirely environmentally friendly. The study has identified three (*n* = 3) types of simple technological interventions for post-stroke rehabilitation, which are telerehabilitation, simple tech assistive technologies (AT), and gait training (GT).

#### Telerehabilitation

Telerehabilitation, commonly referred to as tele-therapy or tele-stroke, is information and telecommunications technology that allows clients to get medical care from doctors located remotely (76). It is one of the distance-supporting therapies to help both survivors and caregivers. Five (*n* = 5) studies (37, 52, 65, 66, 68) were identified in this scoping review explored telerehabilitation as the technological aid to help the clients in rehabilitation. These studies (37, 52, 68) show that the application of telerehabilitation is effective as an adjunct to the rehabilitation process because it is comfortable and easy to be used, which contributes to the stroke recovery process. In addition, telerehabilitation could be a promising solution in stroke rehabilitation since this technology may simulate autonomous repetitive practice to improve the health outcome (65, 66). The telerehabilitation technologies used in the studies were video-based therapy, a smartphone with ECG-recording, and videoconferencing with tele-therapists.

#### Low-Tech Assistive Technology

Equipment used to increase, maintain or improve the functional capabilities of individuals with disabilities in all aspects of life, including at work, home, or in the community-dwelling is referred to as assistive technology (AT) (32, 61). Whereas low-tech assistive technology or low-tech AT is defined as devices or equipment that do not require much training, inexpensive, and do not have complex or mechanical features (32). Low-tech AT differs from high-tech AT because it does not require the use of a power source (36). There are three studies (*n* = 3) (32, 36, 61) identified to use low-tech AT, such as light touch and force contact cane, insole shoe wedges, and a new multidirectional reach tool. The use of this simple technological intervention has shown a progressive effect to improve the stability and balance of stroke survivors. Integration of low-tech AT toward conventional training is suggested to enhance gait speed, standing and walking symmetry, and balance. In addition, low-tech AT is considered an alternative and inexpensive tool for balance training in stroke rehabilitation treatment (61).

#### Gait Training (Treadmill Training)

Gait analysis measures movement in various situations, making it significant and helpful in numerous applications such as rehabilitation activities, sports training, and identifying problems, among others (80). Walking speed, body-weight-bearing abilities, and balance have all improved because of the training. The training allows therapists to track oxygen uptake, which helps the cardiovascular health assessments (94). It aids in treating neurophysiological problems and the stimulation of paretic muscles. Treadmill training was used in two (*n* = 2) studies (49, 54) to assist stroke survivors in their rehabilitation sessions. A variable automated speed and sensing treadmill (VASST) was adopted for chronic stroke by Chua et al. (49) and this technique was proven to be beneficial, safe, and practicable to use. In contrast to another study (54), the combination of electromechanical gait trainers (GT) and conventional therapy has the same effect on stroke survivors' ambulation and health status than conventional therapy alone. As a result, there is no significant difference compared to merely using conventional treatments.

### Stakeholder's Consultation

The stakeholders involved were from two major disciplines i.e., technical sciences (industrial designer, engineer) and health sciences (physiatrist, occupational therapists, and physiotherapist). Findings from the stakeholders' discussion are summarized below.

The current method implemented in stroke rehabilitation or stroke recovery process is mostly conventional methods. The technological intervention is said to be an adjunct to the current conventional therapies where it can be implemented to help stroke survivors to recover faster. In Southeast Asia countries, however, especially in the less developed nations, there is still a lack of technological interventions applied for rehabilitation purposes even in the tertiary hospitals.

In addition, the intervention implemented should be able to motivate the stroke survivors during the rehabilitation session and give meaningful outcome to them. The technological intervention also needs to reduce the labor costs of stroke rehabilitation, while simultaneously allow the survivors to undergo their treatments with minimum supervision. In some cases, however, survivors tend to rely entirely on the interventions and become too dependent on the therapy, which eventually causes other impairments to occur. If this happens, the technological intervention is not much different from the conventional method and could be neglected. The appropriate technological intervention to be implemented should be flexible and not limited to only specific functional impairment and easy to be learned by the practitioners and caregivers.

On the other hand, technological intervention in rehabilitation might bring challenges to both practitioners and survivors. For example, the survivors need to be fully assisted during the rehabilitation session when using the advanced technological intervention at the hospitals. It is preferred for the technological intervention with long and continuous practice to be carried out as a home-based therapy to improve the recover process. The technology-based therapy is recommended to be applicable toward most stroke survivors with various stages or severities, and diverse functional impairments. Moreover, the technology should be intuitive, interesting and attractive in terms of functionality and physical appearance to encourage the continuous use (95).

From the consultation session, it can be concluded that the technological interventions are expected to facilitate the needs of survivors, practitioners, and caregivers during the rehabilitation. The intervention should also be user-friendly, safe to use, and easily operated by the clients and healthcare practitioners. In addition, most of the existing interventions found in Southeast Asia were classified as high-tech interventions, and the clients are required to attend the therapy session in the hospital or rehab center. This adds to the financial burden of the survivors and their families. Therefore, it is highly recommended to make full use of the current advanced technology to help reduce the recovery period and provide a more affordable home-based technological intervention for post-stroke rehabilitation.

## Discussions

This scoping review addresses a new research area related to the technological therapy implemented in stroke rehabilitation for stroke survivors in Southeast Asia. It provides a comprehensive understanding of this topic and has identified the critical gaps. The consultation session conducted with the stakeholders provides additional value to the review. According to a framework designed by Arksey and O'Malley (96), a review can be improved, and the findings generated are more valuable when practitioners and consumers contribute to the work in the consultation session (97).

The main goal of rehabilitation is to help stroke survivors be as independent as possible and attain the best quality of life (98). The treatment receives by the stroke survivors is to help them relearn the lost skills when part of the brain is damaged. These skills may include coordinating the leg movements to regain walking ability or teaching survivors new ways of performing tasks by complementing the remining disabilities (99). There is a strong consensus among the rehabilitation experts that the most important element in any rehabilitation programme is for the stroke survivors to be carefully directed, well-focused, and perform a continuous practice (100).

From the past decade, new technology is constantly changing the rehabilitation arena. Technological treatment is helping in neuroplasticity and learning progress i.e., the key in the recovery process of stroke (94). Moreover, the technological treatment gives clients more repetitions, practice time, and intensity than the conventional method.

### The Lack of Technology Utilization

It is pertinent that many countries in Southeast Asia build rehabilitation centres to benefit in stroke rehabilitation treatment. However, most centres rely on conventional therapy rather than utilizing technology for rehabilitation. The critical factor to the lack of technology utilization in rehabilitation facilities is the dearth of urgency and emphasis for clinical rehabilitation by the policy-making authorities (101). The clients at the rehabilitative phase are often not in dire need than those in emergency trauma or requiring a lifesaving procedure (102). Besides, the lack of awareness among healthcare professionals toward advanced rehabilitation technology may influence the utilization of technology in rehabilitation facilities (103). The absence of active interest groups or society in promoting rehabilitation technology also hinders the awareness of the advantage of advanced rehabilitation equipment. Furthermore, conventional practices and manual therapy have long been accepted by therapists to be providing substantial benefits to clients (104). One of the purposes of using the technological intervention is to reduce the burden of stroke survivors' caregivers and families. However, if the cost of the technology is not proportional to the benefits obtained from its application, it will lead to financial burden for the stroke survivors and their family members. Thus, there is a need to ensure the cost-effectiveness of the interventions implemented for this purpose.

### Country's Economic Status Influences the Implementation of Technological Interventions

Based on this scoping review, Singapore has reported the highest number of studies (*n* = 25) involving the advanced technological intervention implemented in stroke rehabilitation and followed by Thailand (*n* = 10), Malaysia (*n* = 5), and Indonesia (*n* = 1). In Southeast Asia, Singapore has rapidly developed from a low-income country to a high-income country (105) and has become another developed country after Brunei (106). Likewise, Thailand has made extraordinary social and economic development progress during the last four decades, shifting from a low-income to an upper-middle-income country in less than a generation (107). As a result, Thailand has become a reference for a nation's development success story, with robust growth and significant poverty reduction and significant social progress (107).

This paper reveals that the acceptance of technological advancement has a relevant correlation with the country's economic growth. This correlation is apparent when comparing the number of studies using technological interventions in stroke rehabilitation in Southeast Asia countries based on their economic status. It justifies why Singapore has reported the most studies followed by other countries. Unfortunately, in low and middle-income countries, the technology for rehabilitation is not widely used, though there are attempts on its applications for therapeutic purposes (108, 109).

In this scoping review, most of the interventions used were advanced technologies and found mostly in the tertiary hospitals or the rehabilitation units. Besides, the stroke rehabilitation facilities available are platform-based. This could be somewhat inconvenient in terms of traveling and logistics, especially if the client stays far away from the health institutions. Due to this, the home-based therapies should at least offer compatible benefits and affordable in price. An overpriced technological aid causes a burden on survivors' caregivers and their family members, eventually demotivating them to continue the therapy sessions.

### Further Promoting the Use of Technological Interventions in Stroke Rehabilitation

The mentioned technological interventions in the selected studies were mostly practical to be implemented as an adjunct to conventional therapy. However, in this scoping review, it is found that most of the technological interventions applied were focused on physical training, especially on the upper extremities, and only a few studies were meant for the lower extremities. When a person is diagnosed with a stroke, he/she usually experience difficulties in performing the activities of daily living (ADL) due to weakened mobility (110). A review by Hobbs & Artemiadis (111) suggested the exploration of other technologies for lower limb stroke rehabilitation—which were not found in this scoping review, such as on physical implementation (i.e., exoskeleton and powered orthoses) and targeted sensorimotor pathways (i.e., vision and auditory feedback, equilibrioception, cutaneous and haptic perception, inter-limb coordination mechanisms). Hence, it is necessary for the stroke survivors to perform repetitive lower limb exercises to help them regain the gait, balance, and overall mobility. This is, therefore, highlights the need to encourage more studies on technological intervention for lower extremity rehabilitation.

Equally important, more attention on other components such as cognitive, social, and emotional support using technology is required, as agreed by the previous review finding (2). There are several other technological advancements for rehabilitation that can be researched by referring to the international reviews, which include the use of information technology and apps for home-based, cognitive and caregivers' intervention (112–115), wearable devices for upper-limb, participation intervention (116, 117), and other emergence availability of the Industrial Revolution 4.0 and Internet of Things.

Another essential point, technological interventions reported in the reviewed studies mostly took place in hospitals and rehabilitation centres, with very few in the community-dwelling population. Traveling from home to the hospitals regularly for continuous therapy sessions could be burdensome, especially for rural areas (118). More importantly, the current coronavirus (COVID-19) pandemic outbreak causes an urgent need to reduce hospital stays and visits (119). At this crucial time, stroke clients have been forced to be catered to as a lower priority to avoid overburdening the healthcare system (119, 120). Because of this, the clients' therapy sessions have been reduced as a result of physical distancing and this has indirectly affected the quality of care in stroke survivors. For the same reason, the technological intervention should be fundamentally safe, user-friendly, cost-effective, engaging and motivating. The home-based technological aids or devices can also be designed to be portable or even wearable to fit into a limited space. Furthermore, these devices or tools should be durable so that little maintenance is required over potentially long periods of use (119).

It is relevant to use technological intervention as an alternative method to provide clients with high-quality therapy to optimize long-term functional outcomes and promote stroke survivors' independence and quality of life. This is particularly for those who have difficulties traveling to the hospitals or rehabilitation facilities. Therefore, it is undoubtedly that there is an obvious need to further promote the integration of technological aids toward the conventional techniques in stroke rehabilitation, due to positive effect shown on the client's recovery period, a decrease of human labor intensive, reducing the traveling cost, and lessening the hospital visits.

### Limitation

This scoping review has several limitations. Firstly, although the exclusion of gray literature gives a small impact, it may still be beneficial in controlling for the overestimation of conclusion (121), providing better coverage and wider evidence mapping (122). Hence, limited resources in terms of facility and access to gray literature, lack of expertise in searching such evidence, and lack of manpower and financial support have prevented our efforts from including the gray literature. Many publications are made available in English due to the lingua franca status and most of the findings from gray literature were also translated into journal publications for knowledge-sharing purposes (28). Nevertheless, this scoping review is still valuable and comprehensive in conveying the practice.

Secondly, during the stakeholders' consultation, some panels briefly raised the limitation on utilization and acceptance of technology in practice and limited client preferences. Nevertheless, although it is interesting to explore users' perspectives in understanding the use of technology and how it benefits them, it is beyond the scope of this scoping review.

## Conclusion

Strokes cause survivors to live with severe disabilities that affect their daily activities due to paralysis and impaired balance and mobility. From the standpoint of rehabilitation, the clients must engage in an extensive and continuous therapeutic exercise for the recovery process. The conventional stroke rehabilitation techniques usually take longer for stroke survivors to fully recover since they rely on the therapy sessions and exercises conducted by the therapists. New rehabilitation techniques, such as constraint-induced, biofeedback, and robot-assisted therapy, have evolved in recent years and have been embraced as an adjunct to conventional techniques.

The integration of technological intervention toward conventional therapies has shown a positive outcome to the survivor's post-stroke recovery process. This is indeed a better form of therapy especially during the pandemic, where face-to-face consultation is restricted, which at the same time encourages the survivors to undergo treatments. As a result, they could regain independence on mobility and perform the activities of daily living (ADLs). However, there are several gaps identified in this scoping review, which include the lack of studies on technological intervention toward lower extremities. Besides, most of the interventions were found in the hospitals and rehabilitation units, and only a few studies were done in the community-dwelling or home-based therapy. In addition, many technologies are still yet to be explored since this scoping review only covers Southeast Asia countries and the technologies available are predominated by prosperous countries. International references and collaborations should be further encouraged to promote the use latest technological advancements. Nevertheless, this scoping review reveals that the utilization of technologies in stroke rehabilitation has begun to be acknowledged and established in Southeast Asia.

## Data Availability Statement

The original contributions presented in the study are included in the article/Supplementary Material, further inquiries can be directed to the corresponding author/s.

## Author Contributions

The inclusion/exclusion criteria and search strategy were developed under the supervision of MR and RC. The search in the databases was carried out by SS, HA, and MR who also led the writing of the methodology section in the manuscript. SS, RC, HA, and MR worked together on data analysis and manuscript writing. SS, MS, and HR contributed information on the direction of the data analysis and revised continuous amendments of the manuscript draft. The final manuscript was read and approved by all the authors.

## Conflict of Interest

The authors declare that the research was conducted in the absence of any commercial or financial relationships that could be construed as a potential conflict of interest.

## Publisher's Note

All claims expressed in this article are solely those of the authors and do not necessarily represent those of their affiliated organizations, or those of the publisher, the editors and the reviewers. Any product that may be evaluated in this article, or claim that may be made by its manufacturer, is not guaranteed or endorsed by the publisher.

## References

[1] Lindsay MP Norrving B Sacco RL Brainin M Hacke W Martins S . World stroke organization (WSO): global stroke fact sheet 2019. Int J Stroke. (2019) 14:806–17. 10.1177/1747493019881353 31658892

[2] Ahmad Ainuddin H Romli MH Hamid TA Salim MS Mackenzie L. Stroke rehabilitation for falls and risk of falls in Southeast Asia: a scoping review with stakeholders' consultation. Front Public Health. (2021) 9:112. 10.3389/fpubh.2021.611793 33748063 PMC7965966

[3] Ying CY Harith S Ahmad A Mukhali HB. Prevalence, risk factors and secondary prevention of stroke recurrence in eight countries from South, East and Southeast Asia: a scoping review. Med J Malaysia. (2018) 73:90–9. 29703872

[4] WHO Global Health Estimates. WHO. (2018). Available online at: http://www.who.int/healthinfo/global_burden_disease/en/ (accessed March 12, 2021).

[5] Johnson W Onuma O Owolabi M Sachdev S. Stroke: a global response is needed. Bull World Health Organ. (2016) 94:634A−5A. 10.2471/BLT.16.181636 27708464 PMC5034645

[6] Aldehaim AY Alotaibi FF Uphold CR Dang S. The impact of technology-based interventions on informal caregivers of stroke survivors: a systematic review. Telemed e-Health. (2016) 22:223–31. 10.1089/tmj.2015.0062 26274910 PMC5915262

[7] Ying CY Sakinah H Ahmad A Mukhal HB. Prevalence, risk factors and secondary prevention of stroke recurrence in eight countries from South, East and Southeast Asia: a scoping review. Med J Malaysia. (2018) 73:90–9. 29703872

[8] Chongsuvivatwong V Phua K H Yap MT Pocock NS Hashim JH et al. Health and healthcare systems in Southeast Asia: diversity and transitions. Lancet. (2011) 377:429–37. 10.1016/S0140-6736(10)61507-3 21269685 PMC7159068

[9] Donkor ES. Stroke in the 21st century: a snapshot of the burden, epidemiology, and quality of life. Stroke Res Treat. (2018) 2018:1–10. 10.1155/2018/3238165 30598741 PMC6288566

[10] Winstein CJ Stein J Arena R Bates B Cherney LR Cramer SC . AHA/ASA guideline guidelines for adult stroke rehabilitation and recovery. Stroke. (2016) 47:98–169. 10.1161/STR.0000000000000098 27145936

[11] Clare L Bayer A Burns A Corbett A Jones R Knapp M . Goal-oriented cognitive rehabilitation in early-stage dementia: study protocol for a multi-centre single-blind randomised controlled trial (GREAT). Trials. (2013) 14:1–15. 10.1186/1745-6215-14-152 23710796 PMC3680175

[12] Clare L Kudlicka A Oyebode JR Jones RW Bayer A Leroi I . Goal-oriented cognitive rehabilitation for early-stage alzheimer's and related dementias: the GREAT RCT. Health Technol Assess. (2019) 23:1–244. 10.3310/hta23100 30879470 PMC6441850

[13] White MJ Gutierrez A McLaughlin C Eziakonwa C Newman LS White M . A pilot for understanding interdisciplinary teams in rehabilitation practice. Rehabil Nurs. (2013) 38:142–52. 10.1002/rnj.75 23658128

[14] Gittler M Davis AM. Guidelines for adult stroke rehabilitation and recovery. JAMA. (2018) 319:820–1. 10.1001/jama.2017.22036 29486016

[15] Donohue K Hoevenaars R McEachern J Zeman E Mehta S. Home-based multidisciplinary rehabilitation following hip fracture surgery: what is the evidence? Rehabil Res Pract. (2013) 2013:1–10. 10.1155/2013/875968 24455275 PMC3877638

[16] Buhagiar MA Naylor JM Harris IA Xuan W Kohler F Wright R . Effect of inpatient rehabilitation vs a monitored home-based program on mobility in patients with total knee arthroplasty the HIHO randomized clinical trial. J Am Med Assoc. (2017) 317:1037–46. 10.1001/jama.2017.1224 28291891

[17] Doig E Fleming J Kuipers P Cornwell P Khan A. Goal-directed outpatient rehabilitation following TBI: a pilot study of programme effectiveness and comparison of outcomes in home and day hospital settings. Brain Inj. (2011) 25:1114–25. 10.3109/02699052.2011.607788 21902462

[18] Stolee P Lim SN Wilson L Glenny C. Inpatient versus home-based rehabilitation for older adults with musculoskeletal disorders: a systematic review. Clin Rehabil. (2012) 26:387–402. 10.1177/0269215511423279 21971753

[19] Putrino D. Telerehabilitation and emerging virtual reality approaches to stroke rehabilitation. Curr Opin Neurol. (2014) 27:631–6. 10.1097/WCO.0000000000000152 25333603

[20] Ifejika-Jones NL Barrett AM. Rehabilitation-emerging technologies, innovative therapies, and future objectives. Neurotherapeutics. (2011) 8:452–62. 10.1007/s13311-011-0057-x 21706265 PMC3148149

[21] Ferreira B Menezes P. Gamifying motor rehabilitation therapies: challenges and opportunities of immersive technologies. Information. (2020) 11:88. 10.3390/info11020088

[22] Chan CV Kaufman DR. A technology selection framework for supporting delivery of patient-oriented health interventions in developing countries. J Biomed Inform. (2010) 43:300–6. 10.1016/j.jbi.2009.09.006 19796709 PMC2838941

[23] Munn Z Aromataris E Tufanaru C Stern C Porritt K Farrow J . The development of software to support multiple systematic review types: the Joanna Briggs Institute System for the Unified Management, Assessment and Review of Information (JBI SUMARI). Int J Evid Based Healthc. (2019) 17:36–43. 10.1097/XEB.0000000000000152 30239357

[24] Tricco AC Lillie E Zarin W O'Brien KK Colquhoun H Levac D . PRISMA extension for scoping reviews (PRISMA-ScR): checklist and explanation. Ann Intern Med. (2018) 169:467–73. 10.7326/M18-0850 30178033

[25] Peters MD Marnie C Tricco AC Pollock D Munn Z Alexander L . Updated methodological guidance for the conduct of scoping reviews. JBI Evid Synth. (2020) 18:2119–26. 10.11124/JBIES-20-00167 33038124

[26] Young J Forster A. Rehabilitation after stroke. Br Med J. (2007) 334:86–90. 10.1136/bmj.39059.456794.68 17218714 PMC1767284

[27] Nussbaumer-Streit B Klerings I Dobrescu AI Persad E Stevens A Garritty C . Excluding non-English publications from evidence-syntheses did not change conclusions: a meta-epidemiological study. J Clin Epidemiol. (2020) 118:42–54. 10.1016/j.jclinepi.2019.10.011 31698064

[28] Hartling L Featherstone R Nuspl M Shave K Dryden DM Vandermeer B. Grey literature in systematic reviews: a cross-sectional study of the contribution of non-English reports, unpublished studies and dissertations to the results of meta-analyses in child-relevant reviews. BMC Med Res Methodol. (2017) 17:1–1. 10.1186/s12874-017-0347-z 28420349 PMC5395863

[29] Dahlin Ivanoff S Hultberg J. Understanding the multiple realities of everyday life: basic assumptions in focus-group methodology. Scand J Occup Ther. (2006) 13:125–32. 10.1080/11038120600691082 16856469

[30] O'Brien KK Colquhoun H Levac D Baxter L Tricco AC Straus S . Advancing scoping study methodology: a web-based survey and consultation of perceptions on terminology, definition and methodological steps. BMC Health Serv Res. (2016) 16:305. 10.1186/s12913-016-1579-z 27461419 PMC4962390

[31] Ang KK Guan C Sui K Chua G Ang T Kuah C . A clinical study of motor imagery-based brain-computer interface for upper limb robotic rehabilitation. In: Annual International Conference of the IEEE Engineering in Medicine and Biology Society. Singapore: IEEE (2009). p. 5981–4. 10.1109/IEMBS.2009.5335381 19965253

[32] Boonsinsukh R Panichareon L Phansuwan-Pujito P. Light touch cue through a cane improves pelvic stability during walking in stroke. Arch Phys Med Rehabil. (2009) 90:919–26. 10.1016/j.apmr.2008.12.022 19480866

[33] Klaiput A Kitisomprayoonkul W. Increased pinch strength in acute and subacute stroke patients after simultaneous median and ulnar sensory stimulation. Neurorehabil Neural Rep. (2009) 23:351–6. 10.1177/1545968308324227 18981187

[34] Joo LY Yin TS Xu D Thia E Chia PF Kuah CWK . A feasibility study using interactive commercial off-the-shelf computer gaming in upper limb rehabilitation in patients after stroke. J Rehabil Med. (2010) 42:437–41. 10.2340/16501977-0528 20544153

[35] Lambercy O Dovat L Yun H Wee SK Kuah CW Chua KS . Effects of a robot-assisted training of grasp and pronation/supination in chronic stroke: a pilot study. J Neuroeng Rehabil. (2011) 8:63. 10.1186/1743-0003-8-63 22087842 PMC3280186

[36] Sungkarat S Fisher BE Kovindha A. Efficacy of an insole shoe wedge and augmented pressure sensor for gait training in individuals with stroke: a randomized controlled trial. Clin Rehabil. (2011) 25:360–9. 10.1177/0269215510386125 21148267

[37] Redzuan NS Engkasan JP Mazlan M Freddy Abdullah SJ. Effectiveness of a video-based therapy program at home after acute stroke: a randomized controlled trial. Arch Phys Med Rehabil. (2012) 93:2177–83. 10.1016/j.apmr.2012.06.025 22789773

[38] Ang KK Guan C Phua KS Wang C Teh I Chen CW . Transcranial direct current stimulation and EEG-based motor imagery BCI for upper limb stroke rehabilitation. Proc Annu Int Conf IEEE Eng Med Biol Soc EMBS. (2012) 4128–31. 10.1109/EMBC.2012.6346875 23366836

[39] Rajaratnam BS Gui KaiEn J Lee JiaLin K SweeSin K Sim FenRu S Enting L . Does the inclusion of virtual reality games within conventional rehabilitation enhance balance retraining after a recent episode of stroke? Rehabil Res Pract. (2013) 2013:1–6. 10.1155/2013/649561 24024033 PMC3759244

[40] Singh DKA Mohd Nordin NA Aziz NAA Lim BK Soh LC. Effects of substituting a portion of standard physiotherapy time with virtual reality games among community-dwelling stroke survivors. BMC Neurol. (2013) 13:1–7. 10.1186/1471-2377-13-199 24330250 PMC4029492

[41] Tretriluxana J Kantak S Tretriluxana S Wu AD Fisher BE. Low frequency repetitive transcranial magnetic stimulation to the non-lesioned hemisphere improves paretic arm reach-to-grasp performance after chronic stroke. Disabil Rehabil Assist Technol. (2013) 8:121–4. 10.3109/17483107.2012.737136 23244391

[42] Várkuti B Guan C Pan Y Phua KS Ang KK Kuah CWK . Resting state changes in functional connectivity correlate with movement recovery for BCI and robot-Assisted upper-extremity training after stroke. Neurorehabil Neural Repair. (2013) 27:53–62. 10.1177/1545968312445910 22645108

[43] Suriya-Amarit D Gaogasigam C Siriphorn A Boonyong S. Effect of interferential current stimulation in management of hemiplegic shoulder pain. Arch Phys Med Rehabil. (2014) 95:1441–6. 10.1016/j.apmr.2014.04.002 24769123

[44] Yin CW Sien NY Ying LA Chung SFCM Tan May Leng D. Virtual reality for upper extremity rehabilitation in early stroke: a pilot randomized controlled trial. Clin Rehabil. (2014) 28:1107–14. 10.1177/0269215514532851 24803644

[45] Ang KK Guan C Phua KS Wang C Zhou L Tang KY . Brain-computer interface-based robotic end effector system for wrist and hand rehabilitation: results of a three-armed randomized controlled trial for chronic stroke. Front Neuroeng. (2014) 7:1–9. 10.3389/fneng.2014.00030 25120465 PMC4114185

[46] Ang KK Guan C Phua KS Wang C Zhao L Teo WP . Facilitating effects of transcranial direct current stimulation on motor imagery brain-computer interface with robotic feedback for stroke rehabilitation. Arch Phys Med Rehabil. (2015) 96:S79–87. 10.1016/j.apmr.2014.08.008 25721551

[47] Thanakamchokchai J Tretriluxana J Jalayondeja C Pakaprot N. Immediate effects of low-frequency repetitive transcranial magnetic stimulation to augment task-specific training in sub-acute stroke. KKU Res J. (2015) 20:105–19. 10.14456/kkurj.2015.10 PMC6325317 30613070

[48] Ang KK Chua KSG Phua KS Wang C Chin ZY Kuah CWK . A randomized controlled trial of EEG-based motor imagery brain-computer interface robotic rehabilitation for stroke. Clin EEG Neurosci. (2015) 46:310–20. 10.1177/1550059414522229 24756025

[49] Chua KSG Chee J Wong CJ Lim PH Lim WS Hoo CM . A pilot clinical trial on a variable automated speed and sensing treadmill (VASST) for hemiparetic gait rehabilitation in stroke patients. Front Neurosci. (2015) 9:1–9. 10.3389/fnins.2015.00231 26217170 PMC4498099

[50] Samuel GS Choo M Chan WY Kok S Ng YS. The use of virtual reality-based therapy to augment poststroke upper limb recovery. Singapore Med J. (2015) 56:127–30. 10.11622/smedj.2015117 26243983 PMC4520926

[51] Tretriluxana J Kantak S Tretriluxana S Wu AD Fisher BE. Improvement in paretic arm reach-to-grasp following low frequency repetitive transcranial magnetic stimulation depends on object size: a pilot study. Stroke Res Treat. (2015) 2015:1–13. 10.1155/2015/498169 26664827 PMC4664821

[52] Koh GCH Yen SC Tay A Cheong A Ng YS De Silva DA . Singapore tele-technology aided rehabilitation in stroke (STARS) trial: protocol of a randomized clinical trial on tele-rehabilitation for stroke patients. BMC Neurol. (2015) 15:1–4. 10.1186/s12883-015-0420-3 26341358 PMC4560876

[53] Kong KH Loh YJ Thia E Chai A Ng CY Soh YM . Efficacy of a virtual reality commercial gaming device in upper limb recovery after stroke: a randomized, controlled study. Top Stroke Rehabil. (2016) 23:333–40. 10.1080/10749357.2016.1139796 27098818

[54] Chua J Culpan J Menon E. Efficacy of an electromechanical gait trainer poststroke in singapore: a randomized controlled trial. Arch Phys Med Rehabil. (2016) 97:683–90. 10.1016/j.apmr.2015.12.025 26802969

[55] Ahmad MA Singh DK Mohd Nordin NA Hooi Nee K Ibrahim N. Virtual reality games as an adjunct in improving upper limb function and general health among stroke survivors. International journal of environmental research and public health. (2019) 16:5144. 10.3390/ijerph16245144 31888293 PMC6950522

[56] Hong X Lu ZK Teh I Nasrallah FA Teo WP Ang KK . Brain plasticity following MI-BCI training combined with tDCS in a randomized trial in chronic subcortical stroke subjects: a preliminary study. Sci Rep. (2017) 7:1–12. 10.1038/s41598-017-08928-5 28835651 PMC5569072

[57] Samuel GS Oey NE Choo M Ju H Chan WY Kok S . Combining levodopa and virtual reality-based therapy for rehabilitation of the upper limb after acute stroke: pilot study part II. Singapore Med J. (2017) 58:610–7. 10.11622/smedj.2016111 27311739 PMC5651508

[58] Yap HK Lim JH Nasrallah F Yeow CH. Design and preliminary feasibility study of a soft robotic glove for hand function assistance in stroke survivors. Front Neurosci. (2017) 11:547. 10.3389/fnins.2017.00547 29062267 PMC5640819

[59] Sirinuch Utarapichat MD. Effects of transcranial direct current stimulation on motor activity of lower limb muscles in chronic stroke. J Med Assoc Thailand. (2018) 105:131–6.

[60] Tretriluxana J Thanakamchokchai J Jalayondeja C Pakaprot N Tretriluxana S. The persisted effects of low-frequency repetitive transcranial magnetic stimulation to augment task-specific induced hand recovery following subacute stroke: extended study. Ann Rehabil Med. (2018) 42:777–87. 10.5535/arm.2018.42.6.777 30613070 PMC6325317

[61] Khumsapsiri N Siriphorn A Pooranawatthanakul K Oungphalachai T. Training using a new multidirectional reach tool improves balance in individuals with stroke. Physiotherapy Res Int. (2018) 23:e1704. 10.1002/pri.1704 29436087

[62] Utomo B Triwiyanto Suhartini S Luthfiyah S Mudjiono U. Impact of robotic exoskeleton based on electromyography for rehabilitation of post stroke patient. AIP Conf Proc. (2018) 2014:020105. 10.1063/1.505450924313031

[63] Klomjai W Aneksan B Pheungphrarattanatrai A Chantanachai T Choowong N Bunleukhet S . Effect of single-session dual-tDCS before physical therapy on lower-limb performance in sub-acute stroke patients: a randomized sham-controlled crossover study. Ann Phys Rehabil Med. (2018) 61:286–91. 10.1016/j.rehab.2018.04.005 29763676

[64] Foong R Tang N Chew E Chua KSG Ang KK Quek C . Assessment of the efficacy of EEG-based MI-BCI with visual feedback and EEG correlates of mental fatigue for upper-limb stroke rehabilitation. IEEE Trans Biomed Eng. (2020) 67:786–95. 10.1109/TBME.2019.2921198 31180829

[65] Asano M Tai BC Yeo FYT Yen SC Tay A Ng YS . Home-based tele-rehabilitation presents comparable positive impact on self-reported functional outcomes as usual care: the Singapore tele-technology aided rehabilitation in stroke (STARS) randomised controlled trial. J Telemed Telecare. (2021) 27:231–8. 10.1177/1357633X19868905 31462136

[66] Bower KJ Verdonck M Hamilton A Williams G Tan D Clark RA. What factors influence clinicians' use of technology in neurorehabilitation? A multisite qualitative study. Physical Therapy. (2021) 101:pzab031. 10.1093/ptj/pzab031 33522582

[67] Budhota A Chua KS Hussain A Kager S Cherpin A Contu S . Robotic assisted upper limb training post stroke: a randomized control trial using combinatory approach toward reducing workforce demands. Front Neurol. (2021) 12:622014. 10.3389/fneur.2021.622014 34149587 PMC8206540

[68] Koh KT Law WC Zaw WM Foo DH Tan CT Steven A . Smartphone electrocardiogram for detecting atrial fibrillation after a cerebral ischaemic event: a multicentre randomized controlled trial. EP Europace. (2021) 23:1016–23. 10.1093/europace/euab036 33782701

[69] Hu M Cheng HJ Ji F Chong JS Lu Z Huang W . Brain functional changes in stroke following rehabilitation using brain-computer interface-assisted motor imagery with and without tDCS: a pilot study. Front Human Neurosci. (2021) 15:692304. 10.3389/fnhum.2021.692304 34335210 PMC8322606

[70] Luo Z Durairaj P Lau CM Katsumoto Y Do EY Zainuddin AS . Gamification of upper limb virtual rehabilitation in post stroke elderly using silvertune-a multi-sensory tactile musical assistive system. In: 2021 IEEE 7th International Conference on Virtual Reality (ICVR). Singapore: IEEE (2021). p. 149–55.

[71] Mohamad NA Che Adinan SN Yusof Khan AHK Nik Abdul Ghani NNH Kamis MFA-K Wan Sulaiman WA . Transcranial direct current stimulation with multiple oral re-reading therapy for pure alexia without agraphia: a case report. Neurocase. (2021) 3:1–5. 10.1080/13554794.2021.1974487 34478345

[72] Chiri A Vitiello N Giovacchini F Roccella S Vecchi F Carrozza MC. Mechatronic design and characterization of the index finger module of a hand exoskeleton for post-stroke rehabilitation. Trans Mechatr. (2011) 17:884–94. 10.1109/TMECH.2011.214461427295638

[73] Edwards DF Hahn MG Baum CM Perlmutter MS Sheedy C Dromerick AW. Screening patients with stroke for rehabilitation needs: validation of the post-stroke rehabilitation guidelines. Neurorehabil Neural Repair. (2006) 20:42–8. 10.1177/1545968305283038 16467277

[74] Gao D Furukawa K Nakashima H Gao J Wang J Muraoka K. Room temperature deposition of silicon nitride films for passivation of organic electroluminescence device using a sputtering-type electron cyclotron resonance plasma. Japan J Appl Physics. (1999) 38:4868.

[75] Eriksson J Matarić MJ Winstein CJ. Hands-off assistive robotics for post-stroke arm rehabilitation. In: 9th International Conference on Rehabilitation Robotics. Los Angeles, CA: IEEE (2005). p. 21–4.

[76] Poli P Morone G Rosati G Masiero S. Robotic technologies and rehabilitation: new tools for stroke patients' therapy. Biomed Res Int. (2013) 2013:1–8. 10.1155/2013/153872 24350244 PMC3852950

[77] Forrester LW Roy A Krebs HI Macko RF. Ankle training with a robotic device improves hemiparetic gait after a stroke. Neurorehabil Neural Repair. (2011) 25:369–77. 10.1177/1545968310388291 21115945 PMC3565577

[78] De Paula GV Da Silva TR De Souza JT Luvizutto GJ Bazan SGZ Modolo GP . Effect of ankle-foot orthosis on functional mobility and dynamic balance of patients after stroke: study protocol for a randomized controlled clinical trial. Med. (2019) 98:e17317. 10.1097/MD.0000000000017317 31574862 PMC6775434

[79] Bamdadian A Guan C Ang KK Xu J. Online semi-supervised learning with KL distance weighting for Motor Imagery-based BCI. In: 2012 Annual International Conference of the IEEE Engineering in Medicine and Biology Society. Singapore: IEEE (2012). p. 2732–5. 10.1109/EMBC.2012.6346529 23366490

[80] Pollock A Gray C Culham E Durward BR Langhorne P. Interventions for improving sit-to-stand ability following stroke. Cochrane Database Syst Rev. (2014) 1–65. 10.1002/14651858 24859467 PMC6464916

[81] Kumutpongpanich T Senanarong V. Associations between brain imaging characteristics and cognition in post-stroke patients. J Med Assoc Thai. (2017) 100:504–11.28922154

[82] Nurmikko T MacIver K Bresnahan R Hird E Nelson A Sacco P. Motor cortex reorganization and repetitive transcranial magnetic stimulation for pain—a methodological study. Neuro Technol Neural Interface. (2016) 19:669–78. 10.1111/ner.12444 27187056

[83] Thibaut A Moissenet F Di Perri C Schreiber C Remacle A Kolanowski E . Brain plasticity after implanted peroneal nerve electrical stimulation to improve gait in chronic stroke patients: two case reports. NeuroRehabilitation. (2017) 40:251–8. 10.3233/NRE-161410 28222547

[84] Elsner B Kugler J Pohl M Mehrholz J. Transcranial direct current stimulation (tDCS) for improving activities of daily living, and physical and cognitive functioning, in people after stroke. Cochrane Database Syst Rev. (2020) 1–296. 10.1002/14651858.CD009645.pub4 33175411 PMC8095012

[85] Yozbatiran N Keser Z Davis M Stampas A O'Malley M Cooper-Hay C . Transcranial direct current stimulation (tDCS) of the primary motor cortex and robot-assisted arm training in chronic incomplete cervical spinal cord injury: a proof of concept sham-randomized clinical study. NeuroRehabilitation. (2016) 39:401–11. 10.3233/NRE-161371 27589510

[86] Hordacre B. The role of telehealth to assist in-home tDCS: opportunities, promising results and acceptability. Brain Sci. (2018) 8:102. 10.3390/brainsci8060102 29880754 PMC6025370

[87] Ribeiro TH Vieira ML. Motion capture technology—benefits and challenges. Int J Innov Res Technol Sci. (2016) 48:2321–1156.

[88] Kong W Sessa S Cosentino S Zecca M Saito K Wang C . Development of a real-time IMU-based motion capture system for gait rehabilitation. In: 2013 IEEE International Conference on Robotics and Biomimetics (ROBIO). Tokyo: IEEE (2013). p. 2100–5.

[89] Boian RF Deutsch JE Lee CS Burdea GC Lewis J. Haptic effects for virtual reality-based post-stroke rehabilitation. In: *11th Symposium on Haptic Interfaces for Virtual Environment and Teleoperator Systems, 2003. HAPTICS 2003*. Proceedings. New Jersey: IEEE (2003). p. 247–53.

[90] Choi YH Ku J Lim H Kim YH Paik NJ. Mobile game-based virtual reality rehabilitation program for upper limb dysfunction after ischemic stroke. Res Neurol Neurosci. (2016) 34:455–63. 10.3233/RNN-150626 27163250

[91] Mubin O Alnajjar F Jishtu N Alsinglawi B Al Mahmud A. Exoskeletons with virtual reality, augmented reality, and gamification for stroke patients' rehabilitation: systematic review. JMIR Rehabil Assis Technol. (2019) 6:e12010. 10.2196/12010 31586360 PMC6779025

[92] Boian R Lee C Deutsch J Burdea G Lewis J. Virtual reality-based system for ankle rehabilitation post stroke. Workshop Virt Real Rehabil. (2002) 77:86–96.

[93] Shahrin S Rosli A Ab Hadi MH Awang H. A theoretical framework of secure environment of virtual reality application in tertiary TVET education using blockchain technology. J Contemp Soc Sci Educ Studies. (2021) 1:39–46.

[94] Fang Z Yang Z Wang Q Wang C Chen S. A wearable comprehensive data sampling system for gait analysis. J Med Eng Technol. (2018) 42:335–43. 10.1080/03091902.2018.1430184 30324840

[95] Demain S Burridge J Ellis-Hill C Hughes AM Yardley L Tedesco-Triccas L . Assistive technologies after stroke: self-management or fending for yourself? A focus group study. BMC Health Serv Res. (2013) 13:1–2. 10.1186/1472-6963-13-334 23968362 PMC3765821

[96] Arksey H O'Malley L. Scoping studies: towards a methodological framework. Int J Soc Res Methodol. (2005) 8:19–32. 10.1080/1364557032000119616

[97] Oliver S Peersman G Using Research for Effective Health Promotion. Buckingham; Philadelphia, PA: Open University Press (2001).

[98] Kowal M Kołcz A Dymarek R Paprocka-Borowicz M Gnus J. Muscle torque production and kinematic properties in post-stroke patients: a pilot cross-sectional study. Acta Bioengin Biom. (2020) 22:11–20. 10.37190/ABB-01467-2019-02 32307462

[99] Federici S Meloni F Bracalenti M De Filippis ML. The effectiveness of powered, active lower limb exoskeletons in neurorehabilitation: a systematic review. NeuroRehabilitation. (2015) 37:321–40. 10.3233/NRE-151265 26529583

[100] Moreno JC Del Ama AJ de Los Reyes-Guzmán A Gil-Agudo A Ceres R Pons JL. Neurorobotic and hybrid management of lower limb motor disorders: a review. Med Biol Eng Comp. (2011) 49:1119–30. 10.1007/s11517-011-0821-4 21847596

[101] Maggioni S Melendez-Calderon A Van Asseldonk E Klamroth-Marganska V Lünenburger L Riener R . Robot-aided assessment of lower extremity functions: a review. J Neuroeng Rehabil. (2016) 13:1–25. 10.1186/s12984-016-0180-3 27485106 PMC4969661

[102] Tucker MR Olivier J Pagel A Bleuler H Bouri M Lambercy O . Control strategies for active lower extremity prosthetics and orthotics: a review. J Neuroeng Rehabil. (2015) 12:1–30. 10.1186/1743-0003-12-1 25557982 PMC4326520

[103] Gil-Castillo J Alnajjar F Koutsou A Torricelli D Moreno JC. Advances in neuroprosthetic management of foot drop: a review. J Neuroeng Rehabil. (2020) 17:1–9. 10.1186/s12984-020-00668-4 32213196 PMC7093967

[104] Pin TW Butler PB Purves S. Use of whole body vibration therapy in individuals with moderate severity of cerebral palsy-a feasibility study. BMC Neurol. (2019) 19:1–7. 10.1186/s12883-019-1307-5 31043157 PMC6495512

[105] Haberfehlner H Goudriaan M Bonouvrié LA Jansma EP Harlaar J Vermeulen RJ . Instrumented assessment of motor function in dyskinetic cerebral palsy: a systematic review. J Neuroeng Rehabil. (2020) 17:1–2. 10.1186/s12984-020-00658-6 32138731 PMC7057465

[106] Raimi L Olowo R Shokunbi M. A comparative discourse of sustainable finance options for agribusiness transformation in Nigeria and Brunei: implications for entrepreneurship and enterprise development. World J Sci Technol Sustain Dev. (2021) 18:325–50. 10.1108/WJSTSD-05-2021-0051

[107] Estrada G Han X Park D Tian G. Asia's middle-income challenge: an overview, emerging markets finance and trade. (2018). 54:1208–24. 10.1080/1540496X.2017.1421939

[108] Naslund JA Aschbrenner KA Araya R Marsch LA Unützer J Patel V . Digital technology for treating and preventing mental disorders in low-income and middle-income countries: a narrative review of the literature. Lancet Psychiatry. (2017) 4:486–500. 10.1016/S2215-0366(17)30096-2 28433615 PMC5523650

[109] Abaza H Marschollek M. mHealth application areas and technology combinations. Methods Inform Med. (2017) 56(Suppl. 01):e105–22. 10.3414/ME17-05-0003 28925418 PMC6291822

[110] Vourvopoulos A Faria AL Ponnam K Bermudez i Badia S. RehabCity: design and validation of a cognitive assessment and rehabilitation tool through gamified simulations of activities of daily living. In: Proceedings of the 11th Conference on Advances in Computer Entertainment Technology. Madeira: ACM (2014). p. 1–8.

[111] Hobbs B Artemiadis P. A review of robot-assisted lower-limb stroke therapy: unexplored paths and future directions in gait rehabilitation. Front Neuro. (2020) 14:19. 10.3389/fnbot.2020.00019 32351377 PMC7174593

[112] Piran P Thomas J Kunnakkat S Pandey A Gilles N Weingast S . Medical mobile applications for stroke survivors and caregivers. J Stroke Cerebrov Dis. (2019) 28:104318. 10.1016/j.jstrokecerebrovasdis.2019.104318 31416761

[113] Akbari A Haghverd F Behbahani S. Robotic home-based rehabilitation systems design: from a literature review to a conceptual framework for community-based remote therapy during CoViD-19 pandemic. Front Robotics AI. (2021) 8:612331. 10.3389/frobt.2021.612331 34239898 PMC8258116

[114] Chen Y Abel KT Janecek JT Chen Y Zheng K Cramer SC. International journal of medical informatics home-based technologies for stroke rehabilitation. Syst Rev. (2019) 123:11–22. 10.1016/j.ijmedinf.2018.12.001 30654899 PMC6814146

[115] Cogollor JM Rojo-Lacal J Hermsdörfer J Ferre M Waldmeyer MT Giachritsis C . Evolution of cognitive rehabilitation after stroke from traditional techniques to smart and personalized home-based information and communication technology systems: literature review. JMIR Rehabil Assis Technol. (2018) 5:e8548. 10.2196/rehab.8548 29581093 PMC5891670

[116] Maceira-Elvira P Popa T Schmid AC Hummel FC. Wearable technology in stroke rehabilitation: towards improved diagnosis and treatment of upper-limb motor impairment. J Neuroeng Rehabil. (2019) 16:1–8. 10.1186/s12984-019-0612-y 31744553 PMC6862815

[117] Parker J Powell L Mawson S. Effectiveness of upper limb wearable technology for improving activity and participation in adult stroke survivors: systematic review. J Med Int Res. (2020) 22:e15981. 10.2196/15981 31913131 PMC6996755

[118] Chen Y Chen Y Zheng K Dodakian L See J Zhou R . A qualitative study on user acceptance of a home-based stroke telerehabilitation system. Topics Stroke Rehabil. (2020) 27:81–92. 10.1080/10749357.2019.1683792 31682789 PMC7012699

[119] Lambercy O Lehner R Chua KS Wee SK Rajeswaran DK Kuah CW . Neurorehabilitation from a distance: can intelligent technology support decentralized access to quality therapy?. Front Robotics AI. (2021) 8:126. 10.3389/frobt.2021.612415 34026855 PMC8132098

[120] Jordan RE Adab P Cheng K. Covid-19: risk factors for severe disease and death. (2020) 368:1–2. 10.1136/bmj.m1198 32217618

[121] McAuley L Tugwell P Moher D. Does the inclusion of grey literature influence estimates of intervention effectiveness reported in meta-analyses? Lancet. (2000) 356:1228–31. 10.1016/s0140-6736(00)02786-0 11072941

[122] Blackhall K. Finding studies for inclusion in systematic reviews of interventions for injury prevention–the importance of grey and unpublished literature. Injury Prev. (2007) 113:359. 10.1136/ip.2007.017020 17916897 PMC2610605

