# Supplementary material for: The Application of Technological Intervention for Stroke Rehabilitation in Southeast Asia: A Scoping Review With Stakeholders' Consultation
Source: Front Public Health. 2022 Feb 7;9:783565. doi: 10.3389/fpubh.2021.783565 (PMC8858807; doi:10.3389/fpubh.2021.783565)
Supplement: Supplementary file 1 [file Table_1.DOCX]

**Table 1** The characteristics and summary of the included studies

| **Author, Year, Country** | **Objectives** | **Design Setting** | **Participants** | **Interventions** | **Outcome Measure** | **Results** |
| --- | --- | --- | --- | --- | --- | --- |
| Ang et al., 2009, Singapore (28) | To investigate the effects of MI-BCI for upper limb robotic rehabilitation compared to standard robotic rehabilitation. | Clinical study | N = 47  Diagnosis  Hemiparetic stroke | MIT Manus robot motor training in form of video games coupled with a non-invasive EEG-based MI-BCI where impaired limbs need to move towards the goal displayed. | Fugl-Meyer (FM) | Efficacious to restore motor control of the upper limb and more practical compare to standard robotic rehabilitation. |
| Boonsinsukh et al., 2009, Thailand (29) | To examine the effect of a light torch cue provided through a cane on mediolateral (ML) pelvic stability during walking in subjects post-stroke. | Crossover trial | N = 40  Diagnosis  Subacute stroke  Impairment  Right-handed | 2 types of cane were used, light touch contact and force contact. A special adjustable-height cane with a sensor was used to test the amount of vertical force where the subject pushed on the cane and they were ordered to walk at a comfortable speed across a 7-metre walkway while keeping the cane in the non-paretic side. | Averaged peak-to-peak pelvic acceleration, muscle activation of the bilateral tensor fascia latae (TFL), semitendinosus (ST) and vastus medialisis | This enhanced sensory information promoting the activation of weight-bearing muscles resulting in improved stability as the paretic leg supports the body weight. |
| Klaiput & Kitisomprayoonkul, 2009, Thailand (30) | To test the effects of peripheral sensory stimulation on pinch strength in patients with acute and subacute stroke. | A randomized, single-blinded, controlled study | N = 20 Diagnosis  Hemispheric stroke  Impairment  Right-handed | 2 hours of simultaneous electrical stimulation over the median and ulnar nerves at the wrist to the level of appreciating paresthesis | Medical Research Council (MRC), Brunnstrom, Light touch, Proprioception, Modified Ashworth, Action Reach Arm Tests (ARAT) | Peripheral sensory stimulation of the paretic hand may increase pinch strength of acute and subacute stroke patients immediately after stimulation. |
| Joo et al., 2010, Singapore (31) | To assess the feasibility of using the Nintendo Wii as an adjunct to conventional rehabilitation of patients with post-stroke upper limb weakness. | Pilot study | N = 20  Diagnosis Stroke  Impairment  Right-side (moderate) | NW was placed and bring into line to ensure patients were able to point at the sensor with neither the position on top or at the base of the television with Wiimote. | FMA, Motricity Index, MAS and VAS Pain | NW is a practical device to increase the conventional therapy in subacute stroke patients. |
| Lambercy et al., 2011, Singapore (32) | To investigate the feasibility of using the HapticKnob, a table-top end-effector device, for robot-assisted rehabilitation of grasping and forearm pronation/ supination, two important functions for activities of daily living involving the hand, and which are often impaired in chronic stroke patients. | Pilot study | N = 15 Diagnosis  Stroke  Impairment  Arm and hand functions | Impaired arms were placed on the padded support while subjects seat in an upright position and grasp the Haptic Knob with their hand. Velcro bands were used to prevent fingers and thumb from slipping. | Fugl-Meyer Motor Assessment (FM) and Motricity Index (MI) | Haptic Knob is advantageous to be used with patients presenting a large range of impairment levels. |
| Sungkarat et al., 2011, Thailand (33) | To determine whether external feedback promote symmetrically  weight distribution during standing and walking would improve gait performance and balance in people with stroke. | Randomized, controlled, assessor-blinded trial | N = 35  Diagnosis  Stroke Randomly group  Experimental  Control | Insole shoe wedges with sensor set up are used in experiment group and conventional method on the other. | Berg Balance Scale, Timed Up and Go, GAITRite | The rehabilitation on standing and walking symmetry, gait speed and balance were improved compared to the conventional method. |
| Redzuan et al., 2012, Malaysia (34) | To evaluate the effectiveness of an intervention using video to deliver therapy at home for patients with stroke. | Randomized controlled trial | N = 90,  Diagnosis  Acute, first hemiplegic,  Had caregiver program in 3 months study, and discharge patients.  Randomly assigned | Combination of home rehabilitation guided using a digital video disk that has therapy techniques and follows up for intervention group. | Modified Barthel Index (MBI), the incidence of strokes complications and Caregiver Strain Index | Video-based therapy is practical to use as it does not give any negative effect on independence and is agreeable to the caregivers. |
| Ang et al., 2012, Singapore (35) | To investigate the effects of combining tDCS with MI-BCI and robotic feedback compared to sham-tDCS for upper limb stroke rehabilitation. | Clinical study | N =19,  Diagnosis  Hemiparetic stroke | A direct current from a surface sponge electrode was tested on the patients. | Motor cortical excitability | tDCS group shows higher accuracies across 10 sessions of rehabilitation. |
| Rajaratnam et al., 2013, Singapore (36) | To evaluate if interactive virtual reality balance-related games integrated within conventional rehabilitation sessions resulted in more superior retraining of dynamic balance compared to CR after stroke. | Randomized controlled and double-blinded pilot study | N = 19  Diagnosed Stroke, randomly assigned  (n=19) | The experimental group received 40 minutes of conventional therapy and 20 minutes of virtual reality rehabilitation either Nintendo Wii or Microsoft Kinect game console system while the control group received only the conventional method. | Functional Reach Test (FRT), Timed Up and Go (TUG), Modified Barthel Index (MBI), Berg Balance Scale (BBS), and Centre of Pressure (CoP) | Virtual reality is effective to be included with conventional therapy as it was able to keep up the postural standing balance. |
| Singh et al., 2013, Malaysia (37) | To determine whether there were any changes in physical function and activities of daily  livings when substituting a portion of the standard physiotherapy time with virtual reality games among community-dwelling stroke survivors. | Randomised controlled trial | N = 50, Diagnosis  Stroke survivors NASAM group  Age: ≥ 55 | The experimental group received 30 minutes of VR balance games edition as an addition to 90 minutes of standard physiotherapy. The control group received full standard physiotherapy. | Timed Up and Go (TUG) test, 30-second Sit to Stand test (30sSTS), Timed Ten-Metre Walk test, Six-Minute Walk Test and the Barthel Index (BI), and static balance was assessed using a balance board. | By adding standard physiotherapy in the VR games are the same as only applied standard physiotherapy alone. |
| Tretriluxana et al., 2013, Thailand (38) | To investigate the effect of inhibitory low-frequency repetitive Transcranial Magnetic Stimulation (rTMS) applied to  the non-lesioned hemisphere on kinematics and coordination  of paretic arm reach-to-grasp (RTG) actions in individuals with  stroke. | Phase 1 trial (feasibility and efficacy) | N = 9  Diagnosis  Post-stroke  Right-handed dominant  M=5  F=4  Mean age: 59 (6.8) years | Two TMS treatments were performed on two separate days: active rTMS and sham rTMS. Cortico-motor excitability (CE) of the non-lesioned hemisphere, as well as RTG kinematics of the paretic hand as participants reached for a dowel of 1.2 cm in diameter, was assessed before and after the rTMS treatments | Motion Monitor (Innovative Sports Training Inc, Chicago IL), an electromagnetic motion system with 6 degrees of freedom mini-BIRD sensors (Ascension Technologies) | LF-rTMS can enhance the paretic arm reach-to-grasp performance on the non-lesioned hemisphere. |
| Várkuti et al., 2013, Singapore (39) | To examine the effects of upper-extremity robot-assisted rehabilitation (MANUS) versus an EEG-based brain-computer interface setup with Motor Imagery (MI EEG-BCI) and compared pre-treatment and post-treatment RS-fMRI. | Pilot Study | N=9  Diagnosis  Upper extremities  First ischemic or hemiplegic stroke (moderate to severe)  Age: 21-65 years old | Impaired shoulder and elbow muscles that were strapped on the robot-end-effector was moved towards the goal displayed on a video screen. (MANUS), Manus robot connected to the EEG-based MI-BCI and have 2 phases calibration and rehabilitation (MI-BCI) | RS-FRMI, Fugl-Meyer (FM) | Functional connectivity changes (FCCs) may predict the steepness of individual motor gains. MI is a potential facilitator of such neuroplasticity. |
| Suriya-Amarit et al., 2014, Thailand (40) | To study the immediate effects of interferential current stimulation (IFC) on shoulder pain and pain-free passive range of motion (PROM) of the shoulder in people with hemiplegic shoulder pain (HSP). | Double-blinded, placebo-controlled, clinical trial | N=30,  Diagnosis  Hemiplegic shoulder pain | Using a match-paired method, participants were grouped into IFC and placebo where the IFC group received 20 minutes with an amplitude-modulated frequency at 100Hz and the current intensity increased until they felt a strong tingling sensation. | Pain intensity shoulder pain assessed by an 11-point NSR | IFC can help in relieving the pain during the movement and increase the pain-free PROM of shoulder pain in the patient. |
| Yin et al., 2014, Singapore (41) | To investigate the effect of virtual reality (VR) rehabilitation on upper extremity motor performance of patients with early stroke. | A pilot randomized controlled trial | N=23  Diagnosis  Adults with strokes  Randomly group | VR group received nine 30 minutes of upper extremity VR therapy in standing additional with conventional therapy which includes both physical and occupational therapy, whilst the control group received only conventional therapy. | Fugl-Meyer Assessment (FMA), Action Reach Arm Test (ARAT), Motor Activity Log (MAL), Functional Independence Measurements (FIM) | Both the VR group and control group shows an improvement over time but no significant difference was shown. |
| Ang et al., 2014, Singapore (42) | To investigate the efficacy of an Electroencephalography (EEG)-based Motor Imagery (MI) Brain-Computer Interface (BCI) couple with a Haptic Knob (HK) robot for arm rehabilitation in stroke patients. | Randomized controlled trial | N=21  Diagnosis  First clinical chronic hemiplegic stroke  Age: 21-80 years old  Randomly group | A total of 18 therapy sessions were received by all groups. BCI-HK groups received an hour of BCI coupled with HK intervention. HK group received an hour of HK intervention and the SAT group only received a standard therapy session. | Fugl-Meyer Motor Assessment (FMMA) | BCI-HK is practical, risk-free, and can increase motor recovery in chronic stroke when combines with therapists-assisted arm mobilization |
| Ang et al., 2015, Singapore (43) | To investigate the efficacy and effects of transcranial direct current stimulation (tDCS) on motor imagery brain-computer interface (MI-BCI) with robotic feedback for stroke rehabilitation. | Randomized Controlled Trial | N=19  Diagnosis  Chronic stroke  Moderate to severe upper extremity functional impairment | Ten sessions of 20 minutes of tDCS or sham before 1 hour of MI-BCI with robotic feedback upper limb stroke rehabilitation for 2 weeks. | Fugl-Meyer Motor Assessment (FMMA) | The averaged online accuracy of the evaluation part and the averaged ERD laterality coefficient of the therapy part for subjects who underwent tDCS were significantly higher than for those who received sham. Hence, the results suggest a role for tDCS in facilitating MI in stroke. |
| Thanakamchokchai et al., 2015, Thailand (44) | To examine the immediate effects of a single session of LF-rTMS with task-specific training based on CIMT and ASAP principles on the performance of the paretic hand in individuals with sub-acute stroke | Single-blinded clinical trial | N=16,  Diagnosis  First subacute stroke | Both groups underwent task-specific training with the paretic hand and constrained the non-paretic hand by a mitt for 1 hour. The stimulation was delivered via the figure of eight air-cooled coils with Magstim rapid for the experimental group. | Motor evoked potential (MEP) amplitude to evaluate the corticospinal excitability of the non-lesioned hemisphere, total movement time (TMT) of the Wolf Motor Function Test to evaluate the behavioural outcomes of both hands. | A single session of LF-rTMS of non-lesioned hemisphere augmented the task-specific training thereby improved paretic hand dexterity which can be proved by the reduction of MEP amplitude of the non-lesioned hemisphere and TMT of the paretic hand performance. |
| Ang et al., 2015, Singapore (45) | To investigate the efficacy of an EEG based MI BCI system coupled with MIT-Manus shoulder-elbow robotic feedback (BCI-Manus) for subjects with chronic stroke with upper-limb hemiparesis. | Randomized Controlled Trial | N=26,  Diagnosis Hemiplegic stroke  Randomly allocated the subjects | Both groups received a total of 18 hours of intervention delivered for over 4 weeks in presence of OT and an engineer.  1) BCI-Manus that consists of EEG-based MI-BCI with Manus robotic feedback,  2) Manus robotically guided shoulder and elbow reaching exercises with computer screen visual feedback using the clock-face game. | Fugl-Meyer Assessment of Motor Recovery after Stroke (FMMA) | The EEG-based MI-BCI therapy achieved significant motor function improvement. |
| Chua et al., 2015, Singapore (46) | To tests a Variable Automated Speed and Sensing Treadmill (VASST) using a standard clinical protocol. | Feasibility clinical study | N=10,  Diagnosis Chronic stroke | VASST is a semi-automated treadmill with multiple sensors and micro controllers, including wireless control to reposition a fall prevention harness, variable pre-programmed exercise parameters and laser beam foot sensors that were positioned on the belt to detect the subject's foot position. | 10mWT, 6mWT, Berg Balance Scale (BBS), Functional Ambulation Category (FAC) | Uses of VASST for gait training of ambulant chronic stroke patients is harmless, practicable and efficacious. |
| Samuel et al., 2015, Singapore (47) | To illustrate the complementary use of biomechanical and kinematic in-game markers, in addition to standard clinical outcomes, to comprehensively assess and track a patient’s disabilities. | Case report | N=1  Female  Diagnosis  Right-side weakness  Acute ischemic stroke Age: 65 years old | Developed software that will simulate the affected arm that was strapped with an IMU to bring the virtual food items to the mouth. To encourage the elbow flexion and extension of the arm. | Functional Independence Measure (FIM), Upper-Extremity Fugl-Meyer (FM) Scale, Action Research Arm Test (ARAT) | Virtual reality-based therapy has a lot of potential as adjuvant therapy in upper limb stroke recovery and can be employed effectively in an acute inpatient environment. |
| Tretriluxana et al., 2015, Thailand (48) | To investigate the effect of LF-RTMS on kinematics and coordination of the paretic hand reach-to-grasp (RTG) for two objects sizes in chronic stroke. | Pilot study | N=9  Diagnosis  Ischemic or haemorrhagic unilateral stroke,  Right-sided weakness,  Mild to moderate  Age: 20-79 years old | The participants received two TMS conditions: real rTMS and sham rTMS conditions. Before and after the rTMS conditions, cortico-motor excitability (CE) of the nonlesioned hemisphere, RTGkinematics, and coordination were evaluated. | For corticospinal excitability of non-lesioned hemisphere (motor evoked potential (MEP) amplitude), for behavioural outcomes of both hands (total movement time (TMT) of the Wolf Motor Function Test | RTG action can only enhance by the LF-rTMS on a smaller object and not on a bigger object. |
| Koh et al., 2015, Singapore (49) | To determine if a novel  tele-rehabilitation  intervention for the first  three months after stroke admission improves functional recovery compared to usual care | Randomized clinical trial | N=100  stroke  patients  Age ≥ 40 years, | Experimental group  will be receiving a tele-  rehabilitation  intervention for 3  months which includes  exercises 5 days per  week using I-Pad based  system and weekly  video conferencing with tele-therapists. | Jette Late Life Functional and Disability Instrument (LLFDI), gait speed (Time 5-Meter Walk Test), endurance (Two Minute Walk Test), Shah Modified Barthel Index, Activities Specific Balance Confidence Scale, patient self-reported health-related quality-of-life [Euro-QOL (EQ-5D)], health  service utilization (Singapore Stroke Study Health Service Utilization Form) and caregiver reported stress (Zarit  Caregiver Burden Inventory). | Several contributions to the practice of rehabilitation  can be made where it will  test the practicability of the tele-technology aided  rehabilitation intervention  on a sufficient number of  participants to provide a  reasonable basis for confident inference to  clinical practice. Exam for any greater functional improvement in the intervention group is due to better adherence to supervised exercise or independent of better adherence to supervised  exercise. |
| Kong et al., 2016, Singapore (50) | To compare the efficacy of a virtual reality commercial gaming device, Nintendo Wii (NW) with conventional therapy and customary care in facilitating upper limb recovery after stroke. | Randomized, controlled, single-blinded study | N=105  Diagnosis  Non-ambulant individuals, 1-month post-stroke | Subjects were divided into a) NW gaming, b) conventional therapy and c) control group. All groups received an hour of physical and occupational therapy. Both NW gaming and conventional therapy groups received 12 sessions of assigned treatment four times a week for 3 weeks and each session lasts for 60 minutes. | Fugl-Meyer Assessment (FMA), Action Research Arm Test (ARAT), Stroke Impact Scale-upper limb items (SIS-UL), Functional Independence Measurements (FIM), Visual Analogue Scale (VAS) | The 12 sessions of the upper limb exercises via NW gaming or conventional therapy does not enhance the upper limb recovery in the subjects with 6 weeks of strokes onset. |
| Chua et al., 2016, Singapore (51) | To evaluate the longer-term effects of electromechanical gait trainers (GT) combined with conventional physiotherapy on health status, function and ambulation in  people with sub-acute stroke, in comparison to conventional physiotherapy given alone. | Randomized Controlled Trial | N=106 Diagnosis  Non-ambulant, one month stroke patient | GT groups received 20 minutes of GT training and 5 minutes of stance/gait training. The control group received 25 minutes of stance/gait training. Both groups completed 10 minutes of standing and 10 minutes of cycling. | Functional Ambulation Category (FAC), Bartel Index (BI), gait and endurance, Stroke Impact Scale (SIS) | The intervention of GT combined with conventional therapy has the same effect as conventional therapy alone in improving the subject ambulation and health status. |
| Ahmad et al., 2017, Malaysia (52) | To examine the effectiveness of VR games as an adjunct to standard  physiotherapy in improving UL function and general health among stroke survivors. | Clinical trial | (n=36) | The experimental group received 0.5h of virtual reality games in a sitting position supervised by therapists using the Cy-Wee Z game controller and 1.5h of standard physiotherapy exercises. | Fugl-Meyer assessment for upper extremities (FMA-UE),  Wolf motor function test (WMFT), intrinsic motivation inventory (IMI), Lawton of instrumental  activities of daily living (IADL), and stroke impact scale (SIS) | The involvement of VR games as an adjunct to standard physiotherapy exercises was equally beneficial in improving the UL compared to standard physiotherapy alone. |
| Hong et al., 2017, Singapore (53) | To investigate brain plasticity following a combined MI-BCI and tDCS intervention in chronic subcortical  stroke patients with unilateral upper limb disability. | Preliminary study | (n=19), female, age 54.1± 10.8 years old, randomly grouped | (TDCS) current ramped up and down to give the stimulation sensation (MI-BCI) mental imagery reaching task where the motor intention detected using the EEG that will be triggered the stroke-affected arm to move. | Fugl-Meyer Assessment (FMA), Resting motor threshold (RMT), Mathlab | tDCS able to assists neuroplasticity and suggest the potential for processing rehabilitation strategies for stroke patients |
| Geoffrey Sithamparapillai Samuel et al., 2017, Singapore (54) | To evaluate the safety and efficacy of a combination of levodopa and virtual reality (VR)-based therapy for the enhancement of upper limb recovery following acute stroke | Pilot study | N=42  Diagnosis  Acute stroke patients Randomly group | The patient engaged in the virtual reality-based game with two inertial measurement units strapped to his right arm (above the elbow) and forearm (above the wrist). The patient right arm (above the elbow) and forearm (above the wrist) were strapped to the virtual reality-based game with two initial measurement units. | Fugl-Meyer Upper Extremity (FM-UE) assessment, Action Research Arm Test (ARAT) | VR-based therapy and pharmacotherapy are practicable to use for acute stroke patients and to gather objective data for analysis. |
| Yap et al., 2017, Singapore(55) | To design and evaluate a soft robotic glove that can provide hand function assistance using fabric-reinforced soft pneumatic actuators. | Pilot test | N=5  Healthy individuals | A soft wearable hand glove was used by the participants on their affected hands to grasp an empty water bottle and a tin can. | FMA, feedback questionnaire | It is possible to use soft wearable exoskeletons that are more wearable, lightweight, and suitable to be used daily for hand function assistance of stroke survivors during activities of daily living. |
| Utarapichat & Kitisomprayoonkul, 2018, Thailand (56) | To study the effects of tDCS on motor activity of lower limb muscles and gait performance in chronic stroke patients. | A patient blind, crossover, sham-controlled pilot study | N=10 chronic stroke patients | Each patient participated in two stimulation conditions of anodal stimulation (2mA, 10 minutes) and sham stimulation (2mA, 30 seconds) and the sequence of the stimulation is randomly assigned. | Root mean square [RMS] amplitude and median frequency [MF] of the vastus medialis oblique [VMO] and tibialis anterior [TA] muscles of the paretic limb and the Timed Up & Go test [TUG] were measured before and immediately after stimulation. | Interventions were unable to the risen motor activity of VMO and TA muscles and TUG in a chronic stroke patient. |
| Tretriluxana et al., 2018, Thailand (57) | To examine the long-term effects of the low-frequency repetitive transcranial magnetic stimulation (LF-rTMS) combined with task-specific training on paretic hand function following subacute stroke. | Double-blinded matched-pair experimental design | N=16  randomly grouped into two | The experimental group received real rTMS with task-specific training, whereas the control group received sham rTMS with task-specific training. The LF-rTMS was applied over M1 of the non-lesioned hemisphere at the hotspot of the extensor digitorum (ED) muscle. The stimulation was delivered through the figure-eight air-cooled coil with a Magstim Rapid stimulator where as the control group received sham stimulation applied using the same coil placement and same TMS parameter as the experimental group except that the coil was tilted 90°. | Motor evoked potential (MEP) amplitude of EDMI, Wolf Motor Function Test (WMFT-TMT). | The improvement in paretic hand after task-specific training was enhanced by LF-rTMS and need to be conducted for at least 2 weeks. |
| Khumsapsiri, 2018, Thailand (58) | To investigate the effect of training using a new multidirectional reach tool on balance in individuals with stroke. | A single-blind randomized controlled trial | N=16  Age: 38 to 72 years old  Randomly assigned | Participants in the experimental group were trained with the multidirectional reach training for 30 min and conventional physical therapy for 30 min per day, 3 days a week for 4 weeks. Participants in the control group received conventional physical therapy for 30 min per day, 3 days a week for 4 weeks. | The participants in the experimental group show improvement in dynamic balance than the control group. The activity assessment on the Fullerton Advanced Balance scale shows improvement in the experimental group than the conventional group after a month. | The study involves only specific criteria which limited the results to be used generally to all types of strokes. The study only investigated a one-month followed-up period. The sample size of the study was underpowered for a difference in post-stroke duration which may influence the results. |
| Utomo et al., 2018, Indonesia (59) | To train the stroke patient independently using a robotic exoskeleton based on electromyography (EMG) signal. | Experimental research | N=5  Diagnosis  Ischemic stroke | EMG signals were collected on the patients' biceps and patients were asked to move the elbow in flexion and extension motion. | MT-3 (Manual Scale Testing) | The use of the robotic exoskeleton method for rehabilitation of stroke patients was not effective for a short time assessment but more preferred as an observation of the EMG activities during the exercise for the post-stroke patient. |
| Klomjai et al., 2018, Thailand (60) | To investigate whether a single session of dual-tDCS before physical therapy could immediately improve lower-limb function and if the improvement would be greater than physical therapy alone. | A randomized sham-controlled crossover study | N=19  Diagnosis  Sub-acute stroke with lower limb weakness. | The patients were seated and their arms are comfortably supported to receive the stimulation. A DC portable stimulator delivered a direct current through 2 rectangular saline-soaked sponge-pad electrodes with a 35 cm2 surface. A 10–20 electroencephalography system was used to apply anodal tDCS over the M1 of the affected hemisphere and cathodal tDCS over the M1 of the unaffected hemisphere, with the medial border of each electrode, placed 5 mm lateral from the vertex. The current flowed continuously for 20 min during the real condition. As for the conventional group, participants received PT for 1 h under the guidance of a physical therapist with 10 years’ experience in stroke rehabilitation, with blinding to the tDCS intervention. | Timed Up and Go (TUG) and Five-Times-Sit-To-Stand (FTSTS) tests and muscle strength was assessed by peak knee torque of extension. | A single session of dual-tDCS before the conventional physical therapy immediately improved the lower-limb function but not the strength of people with sub-acute stroke. |
| Foong et al., 2020, Singapore (61) | To investigate the efficacy of Neuro style Brain Exercise Therapy Towards Enhanced Recovery (nBETTER) system, an EEG-based Motor Imagery Brain-Computer Interface (MI-BCI) employing visual feedback, for upper-limb rehabilitation, and the presence of EEG correlates of mental fatigue during BCI usage. | Clinical study | N=13, Diagnosis  First ischemic or haemorrhagic stroke  Age: 21-80 years old | (n-BETTER system) a lightweight, inter-connected device that detects the imagination of movement of the stroke patient. (MI-BCI) perform upper-arm kinesthetics MI on the affected side where they need to move the affected arm-hand forward to reach the imaginary target in front repeatedly. | Fugl-Meyer Assessment (FMA) | The interventions coupled with the traditional method are suitable and safe to use in the subacute or chronic stroke population. |
| Asano et al., 2021 (62) | To evaluate the impact of a novel tele-rehabilitation system on self-reported functional outcomes compared to usual care during the first three months after stroke. | A parallel, two-arm, evaluator-blinded, randomised controlled trial | (n = 124) Adult age more than 40 years old stroke patients suffer within 4 weeks | Received both tele-rehabilitation system and rehabilitation programme and difficulty level and minimum range of motion for each exercise was assigned by therapists based on individual need. | Late-Life Function and Disability Instrument  (LLFDI). | Tele-rehabilitation may be a promising solution for providing post-stroke rehabilitation services in Singapore while lowering barriers to conventional rehabilitation after hospital release and promoting greater involvement. |
| Bower et al., 2021 (63) | To investigate factors influencing technology use by clinicians working in neurorehabilitation. | A multisite qualitative study | (n=18) physical and occupational therapists | Using the Zoom video conferencing, Participants were also asked to categorise themselves into one of five levels of technology adopter, ranging from those with conservative or sceptical attitudes toward technology (ie, “Luddite”) to those who develop new ideas about technology or are often the first to try (ie, “Innovator,” “Early Adopter”). | NIL | The clinicians generally perceived the use of technology as having a beneficial role in improving health outcomes as technology can promote the independent repetitive practice and objectively assess and monitor performance. |
| Budhota et al., 2021 (64) | To investigate how a time-matched combinatory with conventional training toward reducing workforce demands. | Randomized controlled trial | (n=44) subacute chronic stroke survivors | Robotic Therapy (RT) group received 60 min robotic therapy, 30 minutes of conventional therapy. Performed point-to-point reach task with H-Man, which incorporated a performance-based adaptive controller. | Fugl-Meyer (FMA), Action Research Arm Test and Grip strength | The intervention is applicable to be used for improving the stroke motor recovery and reduce the therapist's workload which may reduce the human effort and enhanced productivity. |
| Koh et al., 2021 (65) | To determine the diagnostic yield of 30-day smartphone ECG recording compared with 24-h Holter monitoring for detecting AF >_30 s. | the multicentre, open-label study, randomly | (n=203), age more than 55 years old, without known AF, and ischemic or TIA patients. | KardiaMobile (AliveCorVR  , Mountain View, CA, USA) was used with a compatible smartphone to record the ECG. Patients in the intervention group were instructed to use the monitor 3 times a day for 30 days and record their ECG on three sessions in a day. The recorded ECG data will then be transmitted wirelessly to the web-based archive. | ECG-documented AF, usage of anticoagulation therapy | Among patients >_55 years of age with a recent cryptogenic stroke or TIA, a 30-day smartphone ECG recording significantly improved the  detection of AF compared with the standard repeat 24-h Holter  monitoring. There was a significantly higher proportion of patients prescribed oral anticoagulation therapy after the 30-day smartphone monitoring |
| Hu et al., 2021  (66) | To determine the diagnostic yield of 30-day smartphone ECG recording compared with 24-h Holter monitoring for detecting AF >_30 s.  To investigate the brain functional activity and connectivity changes after a 2-week MI-BCI and tDCS combined intervention in 19 chronic subcortical stroke patients. | Randomized controlled trial | (n=42) stroke patients aged from 21-70 years old with moderate to severe motor impairments. | MI-BCI + tDCS group received 20 min real tDCS where the direct current was applied using a saline-soaked pair of surface sponge electrodes while MI-BCI group received only 20 min sham-tDCS where current ramped up and down to give a sensation of simulation to the subjects. | Fugl-Meyer Assessment (FMA) | The combination of MI-BCI and tDCS did not further improve motor function than only applying MI-BCI without tDCS as both groups gained significant motor function improvement with no group differences. |
| Luo et al., 2021 (67) | To develop a smart multi-sensory musical assistive system, called SilverTune, to promote the rehabilitation outcome of elderly post-stroke patients. | Pilot study | (n=18) | Participants were given an additional 15 minutes and they are required to be in a starting position of upright sitting against the backrest, feet supported on the ground and the affected hand holding on to the device. Those who are unable to grip firmly the device were given a medical bandage to help them. | Fugl-Meyer Assessment-Upper Extremity (FMA-UE), Canadian Occupational Performance Measure (COPM) | The SilverTune intervention appears to be feasible as a game-based rehabilitation tool amongst the chronic stroke patients in this pilot study. |
| Mohamad et al., 2021 (68) | To investigate the effect of multiple oral re-reading (MOR) therapy adjunct with transcranial direct current stimulation (tDCS) in improving reading recovery of a 64-year-old patient with pure alexia without agraphia following a stroke. | A case report | (n=1), a 64-year-old patient with pure alexia without agraphia following a stroke. | The patient was blinded to each therapy and underwent seven consecutive sessions of sham tDCS followed by seven consecutive sessions of real tDCS, coupled with 1-hour MOR therapy during each session. | Western Aphasia Battery (WAB) assessment: reading component, a writing component, total marks. | The coupled of MOR with tDCS therapy may accelerate the reading recovery in patients with pure alexia as the patient showed improvement using both sham tDCS and real-tDCS with better reading comprehension, average reading time, and word per minute after real tDCS. |
